# A biomimetic approach for the concise total synthesis of greenwaylactams A–C†

**DOI:** 10.1039/d3ob01001e

**Published:** 2023-07-24

**Authors:** Nicolas Kratena, Matthias Weil, Peter Gärtner

**Affiliations:** a Institute for Applied Synthetic Chemistry, TU Wien Getreidemarkt 9/163 1060 Vienna Austria nicolas.kratena@tuwien.ac.at; b Institute of Chemical Technologies and Analytics, TU Wien Getreidemarkt 9/164 1060 Vienna Austria

## Abstract

A concise, racemic total synthesis of three sesquiterpenoid alkaloids (greenwaylactams A–C) exhibiting an unprecedented 8-membered benzolactam is disclosed. Key transformations of this work include the ring expansion through cleavage of an indole *via* Witkop oxidation, as well as an HFIP mediated cationic cyclisation to build up the pentacyclic carbon skeleton.

## Abstract 2

A concise, racemic total synthesis of three sesquiterpenoid alkaloids (greenwaylactams A–C) exhibiting an unprecedented 8-membered benzolactam is disclosed.

## Graphical abstract

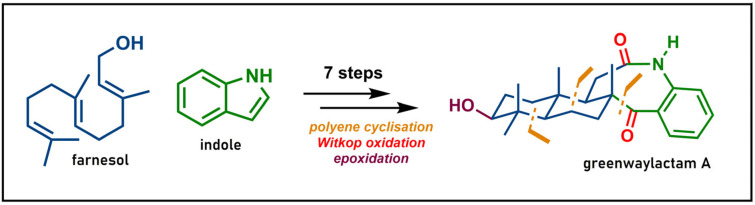

Indole sesquiterpenoids are hybrid natural products (*cf.* meroterpenoids) comprised of an indole moiety as well as a terpenoid framework.^1^ These molecules have attracted a lot of interest due to their complex structural scaffolds and biological activity. Since the discovery of polyalthenol (1) in 1976 more than 200 compounds belonging to this family have been isolated.^2^ However, synthetic efforts have been focused mainly on products containing a C-3 alkylated indole (*cf.*1).^3^ In 2021, the antibacterial natural products greenwaylactams A–C (2–4, see Scheme 1) were isolated from *Greenwayodendron oliveri* by Kouam and Tchamgoue.^4^ The tetracyclic carbon skeleton incorporating an 8-membered lactam immediately attracted our attention. We anticipated that the potential precursor to this lactam could be accessed with exceeding efficiency through a biomimetic polyene cyclisation^5^ of an indole farnesyl derivative.

**Scheme 1:**
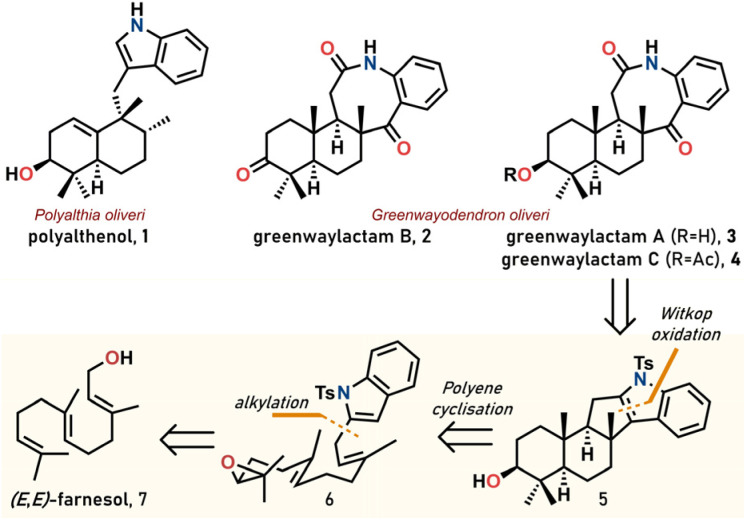
Relevant structures and retrosynthetic analysis of greenwaylactams.

The retrosynthetic analysis started with a Witkop oxidation^7^ of the indole C2–C3 double bond leading back to pentacyclic indole 5. This material was envisaged to be synthesized from an appropriate farnesyl analogue 6 through a cationic cyclisation cascade.^8^ Finally, the required starting material 6 could be obtained after alkylation and epoxidation from commercially available farnesol 7. Indeed, during the preparation of this manuscript, Magauer and co-workers reported their collective total synthesis of greenwaylactams A–C and six related *N*- or *C*-terminated indole sesquiterpenoids (polysin, polyveoline, greenwayodendrins) by polyene cyclisation.^6^

Our synthesis of greenwaylactams started by bromination of 7, to give farnesyl bromide 8 in excellent yield (Scheme 2), which was alkylated with lithiated *N*-tosylindole (10) to give triene 11.

**Scheme 2:**
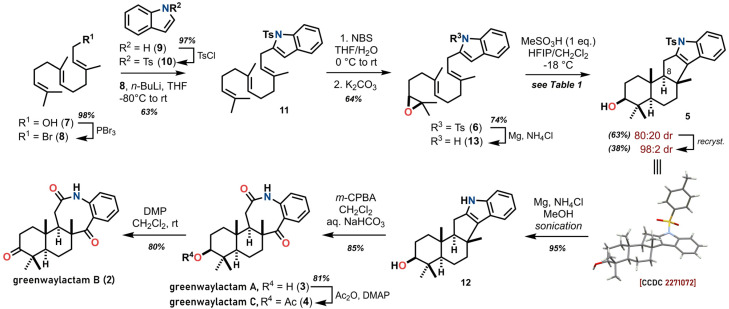
Synthetic route for the total synthesis of greenwaylactams A–C (2–4) from farnesol (7).

Epoxidation of the terminal olefin was achieved in two steps and 64% yield through selective bromohydrin formation by the method of van Temelen^9^ to smoothly deliver epoxide 6, albeit as a racemic mixture. Magauer showed the enantioselective synthesis of a very closely related epoxide, bearing a phenylsulfonyl group instead of a tosylate, could be achieved in a two-step procedure consisting of asymmetric dihydroxylation and mesylation/cyclisation. With the epoxide in hand, the stage was set for the cascade cationic cyclisation which was examined with a range of Lewis or Brønsted acidic conditions as well as by radical initiation in the form of Ti(iii)^10^ (see Table 1). Although many Lewis acids were able to mediate the desired reaction to some extent, after extensive experimentation a mixture of hexafluoroisopropanol^11^ (HFIP) and dichloromethane was found to be optimal. Thus, the desired pentacyclic alcohol 5 was obtained in 63% yield and as a 4 : 1 mixture of diastereomers (minor diastereomer epimeric at C-8, could not be obtained in a pure form, see ESI†). To our delight, the major diastereomer could be easily isolated in 38% yield by recrystallisation and its identity confirmed by single-crystal X-ray diffraction analysis (see Scheme 2).^12^ This bears well in comparison with the results by Magauer's group, who opted for near-freezing HFIP (−4 °C) in combination with catalytic amounts of methanesulfonic acid, obtaining comparable yields and a slightly enhanced diastereoselectivity (67%, dr = 5.7 : 1).^6^ The beneficial effect of fluorinated solvents like HFIP can be rationalised from their excellent hydrogen bond donating abilities, greatly increasing the availability of protons and stabilisation of carbocations.^11^ This effect was not observed with the less acidic 2,2,2-trifluoroethanol (TFE, see Table 1, entry 9).

**Table 1:** Optimisation of conditions for the cationic cyclisation of epoxide 6

#	Reagent^a^	Solvent	Temp.	Time	Yield^b^	dr
1	Et_2_AlCl	CH_2_Cl_2_	−78 °C	0.8 h	40%	67 : 33
2	BF_3_–OEt_2_	CH_2_Cl_2_	−78 °C	1 h	16%^8^	—
3	Cp_2_TiCl	THF	25 °C	2 h	0%	—
4	CF_3_CO_2_H	CH_2_Cl_2_	0 °C	1 h	0%	—
5	InBr_3_	CH_2_Cl_2_	−17 °C	1 h	26%	79 : 21
6	SnCl_4_	CH_2_Cl_2_	−78 °C	1 h	46%	64 : 36
7	MeSO_3_H	HFIP	0 °C	0.2 h	54%	78 : 22
8	MeSO_3_H	CH_2_Cl_2_	−35 °C	0.2 h	Trace	—
9	MeSO_3_H	HFIP/TFE^c^	−20 °C	0.2 h	Trace	—
10	MeSO_3_H	HFIP/CH_2_Cl_2_ c	−18 °C	0.2 h	63%	4 : 1

a 1.0 eq. of reagent was used.

b Referring to isolated yield.

c 1/1 volume ratio.

Some improvements were achieved during the endgame of the synthesis. For instance, the removal of the tosylate protecting group was realised through magnesium mediated reduction in methanol^13^ in excellent yield to give 12. As per the proposed biosynthetic pathway of greenwaylactam A (3) the 5-membered indole ring was then ruptured in 85% yield by Witkop oxidation,^14^ in this case by employing aqueous buffered *m*-CPBA. It was expected that related natural products 4 and 2 would be accessible from greenwaylactam A (3) directly. In our hands, the oxidation of the C-3 alcohol in 3 with Dess–Martin periodinane (DMP) proceeded smoothly to give greenwaylactam B (2) in 80% yield. The C-3 acetylation of 3 was similarly straightforward using 4-DMAP and acetic anhydride providing greenwaylactam C (4) in 81% yield. This improved protocol for C-3 acylation should prove useful for the synthesis of potential analogues of these alkaloids. Gratifyingly, all the analytical data of our synthetic natural products were in agreement with the data obtained in the isolation report^4^ (largest NMR shift deviation: Δ*δ*_H_ = 0.05 ppm, Δ*δ*_C_ = 0.4 ppm, *J*-values match throughout; melting points: 274 *vs.* 277 °C for 3 and 247–250 *vs.* 249–250 °C for 4, for detailed comparison see ESI†) and total synthesis.^6^ Cleavage of the tosyl group before the cyclisation gave rise to 13, which was examined for a potential N-terminated cyclisation. Since a satisfactory solution to this challenging N-termination was already disclosed in the recent report^6^ we decided to conclude our efforts in this synthetic campaign.

## Conclusions

Starting from commercially available starting materials farnesol (7) and indole (9), the three indole meroterpenoids greenwaylactams A–C (2–4) were successfully synthesized in 7 or 8 steps longest linear sequence and in 9–12% overall yield as racemates. The key steps include a selective epoxidation, a polyene cyclisation cascade enabled by the polar non-nucleophilic solvent HFIP and a mild Witkop oxidation of the indole core.

## Author contributions

N. K. conceived of the project, obtained funding, carried out the experiments and contributed to the manuscript. M. W. performed X-ray crystallographic experiments and calculations. P. G. supervised the project and contributed to the manuscript.

## Conflicts of interest

There are no conflicts of interest to declare.

## Supplementary Material

OB-021-D3OB01001E-s001

OB-021-D3OB01001E-s002
