# Supplementary material for: A biomimetic approach for the concise total synthesis of greenwaylactams A–C
Source: Org Biomol Chem. 2023 Jul 24;21(31):6317–9. doi: 10.1039/d3ob01001e (PMC10410498; doi:10.1039/d3ob01001e)

Electronic Supplementary Material (ESI) for Organic & Biomolecular Chemistry.
This journal is © The Royal Society of Chemistry 2023

A biomimetic approach for the concise total synthesis of greenwaylactams A-C

Nicolas Kratena*[1], Matthias Weil[2], Peter Gärtner[1]

[1] Institute for Applied Synthetic Chemistry, Getreidemarkt 9, 1060, Vienna, Austria

[2]Institute for Chemical Technologies and Analytics, Getreidemarkt 9, 1060, Vienna, Austria

Supplementary information

Inhalt

General Experimental Techniques 2
Chemicals and solvents 2
Glassware and reaction conditions 2
Analytical techniques 2
Chromatography 3
X-ray crystallography 3
High-resolution mass spectrometry 3
Procedures 4
(2*E*,6*E*)-1-Bromo-3,7,11-trimethyl-2,6,10-dodecatriene (**8**) 4
1-(*p*-Toluenesulfonyl)indole (**10**) 4
1-Tosyl-2-((2*E*,6*E*)-3,7,11-trimethyldodeca-2,6,10-trien-1-yl)-1H-indole (**11**) 5
2-((2*E*,6*E*)-9-(3,3-dimethyloxiran-2-yl)-3,7-dimethylnona-2,6-dien-1-yl)-1-tosyl-1H-indole (**6**) 6
2-((2*E*,6*E*)-9-(3,3-dimethyloxiran-2-yl)-3,7-dimethylnona-2,6-dien-1-yl)-1-tosyl-1H-indole (**13**)... 7
(3S,4aR,6aR,12aR,12bR)-4,4,6a,12b-tetramethyl-11-tosyl-1,2,3,4,4a,5,6,6a,11,12,12a,12b-dodecahydrobenzo[4,5]indeno[2,1-b]indol-3-ol (**5**) 8
(3S,4aR,6aR,12aR,12bR)-4,4,6a,12b-tetramethyl-1,2,3,4,4a,5,6,6a,11,12,12a,12b-dodecahydrobenzo[4,5]indeno[2,1-b]indol-3-ol (**12**) 9
Greenwaylactam A, (2aR,4S,6aS,6bR,14aR)-4-hydroxy-3,3,6a,14a-tetramethyl-1,2a,3,4,5,6,6a,6b,7,14a-decahydrobenzo[b]naphtho[2,1-e]azocine-8,14(2H,9H)-dione (**3**)........ 10
Greenwaylactam C, (2aR,4S,6aS,6bR,14aR)-3,3,6a,14a-tetramethyl-8,14-dioxo-1,2,2a,3,4,5,6,6a,6b,7,8,9,14,14a-tetradecahydrobenzo[b]naphtho[2,1-e]azocin-4-yl acetate (**4**) 13
Greenwaylactam B, (2aR,6aS,6bR,14aR)-3,3,6a,14a-tetramethyl-1,2a,5,6,6a,6b,7,14a-octahydrobenzo[b]naphtho[2,1-e]azocine-4,8,14(2H,3H,9H)-trione (**2**) 16
References 19

Spectra 20
(2*E*,6*E*)-1-Bromo-3,7,11-trimethyl-2,6,10-dodecatriene (**8**) 20
1-(*p*-Toluenesulfonyl)indole (**10**) 21
2-((2*E*,6*E*)-9-(3,3-dimethyloxiran-2-yl)-3,7-dimethylnona-2,6-dien-1-yl)-1-tosyl-1H-indole (**6**)... 22
2-((2*E*,6*E*)-9-(3,3-dimethyloxiran-2-yl)-3,7-dimethylnona-2,6-dien-1-yl)-1-tosyl-1H-indole (**6**)... 23
(3S,4aR,6aR,12aR,12bR)-4,4,6a,12b-tetramethyl-11-tosyl-1,2,3,4,4a,5,6,6a,11,12,12a,12b-dodecahydrobenzo[4,5]indeno[2,1-b]indol-3-ol (**5**) 24
2-((2*E*,6*E*)-9-(3,3-dimethyloxiran-2-yl)-3,7-dimethylnona-2,6-dien-1-yl)-1-tosyl-1H-indole (**13**). 25
4,4,6a,12b-tetramethyl-1,2,3,4,4a,5,6,6a,11,12,12a,12b-dodecahydrobenzo[4,5]indeno[2,1-b]indol-3-ol (**12**) 26
Greenwaylactam A, 4-hydroxy-3,3,6a,14a-tetramethyl-1,2a,3,4,5,6,6a,6b,7,14a-decahydrobenzo[b]naphtho[2,1-e]azocine-8,14(2H,9H)-dione (**3**) 27
Greenwaylactam C, 3,3,6a,14a-tetramethyl-8,14-dioxo-1,2,2a,3,4,5,6,6a,6b,7,8,9,14,14a-tetradecahydrobenzo[b]naphtho[2,1-e]azocin-4-yl acetate (**4**) 28
Greenwaylactam B, 3,3,6a,14a-tetramethyl-1,2a,5,6,6a,6b,7,14a-octahydrobenzo[b]naphtho[2,1-e]azocine-4,8,14(2H,3H,9H)-trione (**2**) 29

General Experimental Techniques

Chemicals and solvents

Unless stated otherwise, all chemicals were purchased from commercial suppliers (Sigma-Aldrich, BLDPharm, TCI, abcr, Acros, Fisher, VWR) and used without further purification. Some dry solvents (toluene, CH_2Cl_2, THF, Et_2O) were obtained from a PureSolv SPS system by Innovative Technologies. MeCN, MeOH, pyridine, MTBE and all other dry solvents were obtained from Acros Organics over molecular sieves and used without further purification. All other solvents used were HPLC grade or p.a. unless stated otherwise.

Glassware and reaction conditions

Reactions were carried out in round bottom flasks, or oven-dried Schlenk flasks under an inert atmosphere (Argon) unless stated otherwise.

Analytical techniques

^{1}H, and ^{13}C NMR spectra were recorded on a Bruker AVIII 400 Spectrometer (^{1}H: 400 MHz and ^{13}C: 101 MHz) or a Bruker Avance III 600 (^{1}H: 600 MHz and ^{13}C: 151 MHz) in $CDCl_3$, DMSO-*d*$_6$, C_6D_6 or CD_3OD and referenced to residual solvent peaks. Chemical shifts δ are quoted in parts per million (ppm) to the nearest 0.01 for ^{1}H and 0.1 for ^{13}C, coupling constants *J* are quoted in Hz to the nearest 0.1 and splitting are recorded as singlet (s), doublet (d), triplet (t), quartet (q), pentet (p), hexet (h), heptet (hept), and

multiplet (m). Assignments were based upon COSY, HSQC and HMBC experiments. Where unambiguous assignments could not be made the candidate positions are indicated by solidus "/". Due to very small quantities of product in late-stage experiments (<1 mg) residual grease or solvent was observed in some of the NMR spectra. Any grease or residual solvent impurity will be indicated in the spectrum.

Chromatography

Analytical thin layer chromatography was performed on pre-coated silica gel aluminium sheets from Merck (TLC Silica Gel 60 F_{254}). Spots were visualized either by the quenching of UV fluorescence or by staining with phosphomolybdic acid/cerium sulfate, potassium permanganate or acidic *p*-anisaldehyde or vanillin solutions. Preparative column chromatography was carried out using Geduran Silica Gel 60 (40 µm – 63 µm) from Merck. In cases where mixtures of solvents were used, the ratios refer to the component volumes. In cases where gradients where used, the start and the end ratio are stated.

X-ray crystallography

Suitable single crystals were preselected under a polarizing microscope, embedded in perfluorinated polyether and mounted on MiTeGen® loops. The single crystal diffraction measurement was performed on a STOE Stadivari four-circle diffractometer equipped with an Eiger 1M CdTe detector. Intensity data were collected at 100 K using graphite monochromatized Cu *K*α radiation (λ = 1.54186 Å). Correction for absorption effects were carried out with the multi-scan approach of LANA [1]. The crystal structure was solved by using the dual-space algorithm in SHELXT [2] and was refined by the full-matrix least-squares technique on F^2 with SHELXL [3]. H atoms were positioned geometrically (C–H = 0.95-1.00 Å) and were refined as riding with $U_{iso}(H) = 1.2U_{eq}(C)$ for aromatic and methine H atoms, and with $U_{iso}(H) = 1.5U_{eq}(C)$ for methyl H atoms.

CCDC 2271072 contains the supplementary crystallographic data for this paper. These data can be obtained free of charge from The Cambridge Crystallographic Data Centre via www.ccdc.cam.ac.uk/structures

High-resolution mass spectrometry

The HR-MS analysis was carried out from methanol or acetonitrile or water or a mixture of these solvents (concentration: 10 µM) by using an Agilent G7167B multi sampler, an Agilent G7120A binary pump with degasser, an Agilent G7116B oven and Agilent 6545 Q-TOF mass spectrometer equipped with a dual AJS ion score.

Procedures

(2*E*,6*E*)-1-Bromo-3,7,11-trimethyl-2,6,10-dodecatriene (8)

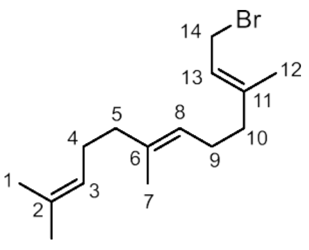

In a 250-mL round-bottom flask was charged *E*,*E*-farnesol (**7**, 5.60 g, 25.2 mmol, 1.0 eq.) in 50 mL of dry diethyl ether. To the solution was added PBr_3 (1.20 mL, 12.6 mmol, 0.5 eq.) dropwise at room temperature and the reaction mixture stirred for 60 minutes. The reaction was quenched by the addition of distilled water (30 mL) and extracted with hexanes. The pooled organic fractions (ca. 150 mL) were dried over $MgSO_4$ and evaporated to give 7.12 g (99% yield) of clean bromide **8**. Analytical data is in accordance with literature [4].

^{1}H NMR (600 MHz, $CDCl_3$) δ = 5.53 (ddt, *J* = 8.5, 7.2, 1.3, 1H, H-13), 5.13 – 5.05 (m, 2H, H-8, H-3), 4.03 (d, *J* = 8.4, 2H, H-14), 2.16 – 2.03 (m, 6H, H-10/H-9/H-5/H-4), 2.00 – 1.94 (m, 2H, H-10/H-9/H-5/H-4), 1.73 (d, *J* = 1.4, 3H, H-12), 1.68 (d, *J* = 1.3, 3H, H-7), 1.60 (s, 6H, H-1).
^{13}C NMR (151 MHz, $CDCl_3$) δ = 143.8, 135.8, 131.5, 124.4, 123.5, 120.7, 39.8, 39.6, 29.9, 26.8, 26.2, 25.9, 17.8, 16.2, 16.1.
HR-MS (ESI): Calculated for $C_{15}H_{25}$ $[M-Br]^+$ 205.1951, found: 205.1956.

1-(*p*-Toluenesulfonyl)indole (10)

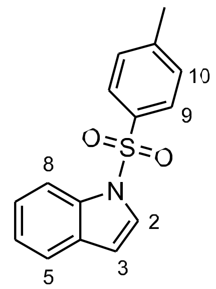

In a 250-mL three-necked flask was charged NaH (ca. 60%, 768 mg, 19.2 mmol, 1.5 eq.) followed by 30 mL dry THF. To the suspension indole **9** (1.50 g, 12.8 mmol, 1 eq.) was added as a solution in 30 mL dry THF over 10 minutes. After stirring the resulting solution for 30 minutes tosyl chloride (2.69 g, 14.1 mmol, 1.1 eq.) in 15 mL dry THF was added via syringe and the reaction was allowed to stir for 16 hours at room temperature. Reaction was quenched with 50 mL water and extracted 3x with EtOAc (250 mL total), to give 3.77 g of crude product. This was purified via flash chromatography (40 g SiO_2, LP:EA = 10:1) to give 3.40 g (98% yield) of 1-tosylindole **10** as a white solid.
Analytical data is in accordance with literature [5].

m.p.: 79 °C (Lit [5]: 78 – 80 °C)
^{1}H NMR (400 MHz, $CDCl_3$) δ = 7.99 (dd, *J* = 8.3, 0.9, 1H, H-5), 7.80 – 7.71 (m, 2H, H-9), 7.56 (d, *J* = 3.7, 1H, H-2), 7.54 – 7.48 (m, 1H, H-8), 7.30 (ddd, *J* = 8.4, 7.2, 1.3, 1H, H-7), 7.24 – 7.18 (m, 3H, H-10, H-6), 6.65 (dd, *J* = 3.7, 0.8, 1H, H-3), 2.34 (s, 3H, CH_3).
^{13}C NMR (101 MHz, $CDCl_3$) δ = 145.1, 135.4, 134.9, 130.9, 130.0 (2C), 126.9 (2C), 126.4, 124.7, 123.4, 121.5, 113.6, 109.2, 21.7.
HR-MS (ESI): Calculated for $C_{15}H_{13}NO_2S$ $[M+H]^+$ 272.0740, found: 272.0740.

1-Tosyl-2-((2*E*,6*E*)-3,7,11-trimethyldodeca-2,6,10-trien-1-yl)-1H-indole (11)

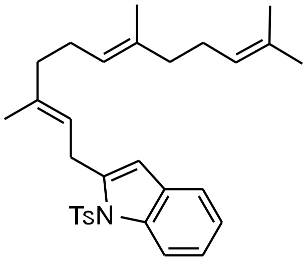

In a Schlenk flask was charged 1-(*p*-toluenesulfonyl)indole (**10**, 998 mg, 3.68 mmol, 1.05 eq.) and dissolved in 20 mL of dry THF. The solution was chilled to -80 °C and n-Butyllithium in hexanes (1.6 M, 2.41 mL, 3.86 mmol, 1.10 eq.) was added dropwise. After 35 minutes of stirring at the same temperature a solution of farnesyl bromide (**8**, 1.00 g, 3.51 mmol, 1.0 eq.) was introduced and the mixture allowed to warm to -40 °C over 2 hours. The cooling bath was then removed, and the reaction allowed to stir at room temperature for an additional hour. Saturated NH_4Cl solution was added to quench the reaction and extractive workup was performed with Et_2O (3x 50 mL). The pooled organic phases were washed with brine, dried over $MgSO_4$ and evaporated. The crude product was purified on 50 g SiO_2 using 40% toluene in hexanes to give 1.05 g (63% yield) of farnesyl indole **11** as a colorless oil. Analytical data is in accordance with literature [6].

^{1}H NMR (600 MHz, $CDCl_3$) δ = 8.18 (dd, *J* = 8.3, 0.9, 1H), 7.65 (d, *J* = 8.4, 2H), 7.39 (dt, *J* = 7.6, 1.0, 1H), 7.25 – 7.23 (m, 1H), 7.23 – 7.12 (m, 3H), 6.35 (d, *J* = 1.0, 1H), 5.41 (td, *J* = 7.2, 1.3, 1H), 5.16 (t, *J* = 6.1, 1H), 5.11 (ddt, *J* = 7.0, 5.6, 1.4, 1H), 3.68 (d, *J* = 7.2, 2H), 2.34 (s, 3H), 2.21 – 2.14 (m, 2H), 2.12 – 2.06 (m, 4H), 2.02 (dd, *J* = 9.2, 6.1, 2H), 1.67 (s, 3H), 1.62 (s, 3H), 1.62 (s, 3H), 1.59 (s, 3H).

^{13}C NMR (101 MHz, $CDCl_3$) δ = 144.8, 141.3, 138.7, 137.5, 136.5, 135.4, 131.5, 129.9 (2C), 129.8, 126.4 (2C), 124.4, 124.1, 123.9, 123.5, 120.2, 119.6, 114.8, 109.0, 39.9, 39.8, 27.9, 26.9, 26.5, 25.8, 21.7, 17.8, 16.2, 16.2.

HR-MS (ESI): Calculated for $C_{30}H_{37}NO_2S$ $[M+H]^+$ 476.2618, found: 476.2622.

2-((2*E*,6*E*)-9-(3,3-dimethyloxiran-2-yl)-3,7-dimethylnona-2,6-dien-1-yl)-1-tosyl-1H-indole (6)

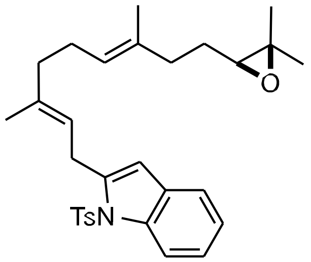

In a 100-mL round-bottom flask was charged triene **11** (750 mg, 1.58 mmol, 1.0 eq.), dissolved in 33 mL of THF/H_2O = 10/1 and stirred in an ice-bath at 0 °C. N-Bromosuccinimide (322 mg, 1.81 mmol, 1.15 eq.) was added as a solid in one portion and the mixture stirred at room temperature for 30 minutes. The reaction was quenched by addition of $NaHCO_3$ solution and extraction with DCM (2x 40 mL). The combined organic phases were dried over $MgSO_4$ and evaporated to give crude bromohydrin **SI-1** (1.07 g). The crude material was dissolved in 40 mL of methanol and K_2CO_3 (651 mg, 4.72 mmol, 3.0 eq.) was added. After stirring at room temperature for 2 hours no bromohydrin could be detected on TLC and the reaction mixture was diluted with Et_2O and H_2O. Extracted 3x with diethyl ether, organic layers dried over $MgSO_4$ and evaporated to give crude product. The crude product was purified on 30 g SiO_2 using hexanes/EA = 10/1 to give 498 mg (64% yield) of epoxide **6** as a viscous oil.

^{1}H NMR (600 MHz, $CDCl_3$) δ = 8.17 (dt, *J* = 8.4, 0.9, 1H), 7.65 (d, *J* = 8.4, 2H), 7.39 (dd, *J* = 8.0, 1.2, 1H), 7.24 (dd, *J* = 8.4, 1.4, 1H), 7.21 – 7.16 (m, 3H), 6.33 (s, 1H), 5.40 (t, *J* = 7.2, 1H), 5.20 (t, *J* = 6.6, 1H), 3.68 (d, *J* = 7.2, 2H), 2.71 (t, *J* = 6.3, 1H), 2.34 (s, 3H), 2.24 – 2.13 (m, 3H), 2.10 (m, *J* = 7.8, 3H), 1.64 (s, 3H), 1.62 (s, 3H), 1.29 (s, 3H), 1.25 (s, 3H).

^{13}C NMR (151 MHz, $CDCl_3$) δ = 144.8, 141.3, 138.6, 137.5, 136.5, 134.5, 130.0, 129.8 (2C), 126.4 (2C), 124.8, 123.9, 123.5, 120.2, 119.7, 114.8, 109.0, 64.3, 58.5, 39.7, 36.5, 27.9, 27.6, 26.6, 25.0, 21.7, 18.9, 16.2, 16.2.

HR-MS (ESI): Calculated for $C_{30}H_{37}NO_3S$ $[M+H]^+$ 492.2567, found: 492.2566.

2-((2*E*,6*E*)-9-(3,3-dimethyloxiran-2-yl)-3,7-dimethylnona-2,6-dien-1-yl)-1H-indole (13)

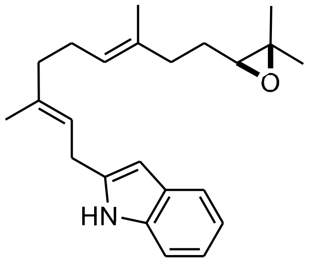

In a 100-mL round-bottom flask was charged epoxide **6** (450 mg, 0.92 mmol, 1.0 eq.) and solid NH_4Cl (979 mg, 18.3 mmol, 20.0 eq.), followed by methanol (20 mL). To the suspension were added magnesium turnings (888 mg, 36.6 mmol, 40.0 eq.) and the reaction mixture was sonicated for 20 minutes (resulted in warming to 35 °C in the water bath). Afterwards the flask was stirred at room temperature for an additional 40 minutes. At that point the reaction was quenched by the addition of NH_4Cl solution and extracted with DCM (3x 30 mL). The crude product after drying with $MgSO_4$ was purified on 27 g SiO_2 using hexanes/EA = 10/1 as eluent to give 228 mg (74% yield) of indole **13** as a yellow oil. Analytical data is in accordance with the literature [7].

^{1}H NMR (600 MHz, $CDCl_3$) δ = 8.01 (s, 1H), 7.52 (dd, *J* = 7.7, 1.1, 1H), 7.30 (dd, *J* = 8.0, 1.0, 1H), 7.10 (ddd, *J* = 8.1, 7.1, 1.3, 1H), 7.08 – 7.01 (m, 1H), 6.23 (d, *J* = 1.1, 1H), 5.44 – 5.37 (m, 1H), 5.23 – 5.13 (m, 1H), 3.50 (d, *J* = 7.2, 2H), 2.72 (t, *J* = 6.2, 1H), 2.23 – 2.14 (m, 3H), 2.14 – 2.07 (m, 3H), 1.74 (s, 3H), 1.70 – 1.62 (m, 2H), 1.64 (s, 3H), 1.30 (s, 3H), 1.26 (s, 3H).

^{13}C NMR (151 MHz, $CDCl_3$) δ = 138.7, 138.1, 136.1, 134.5, 129.0, 124.8, 121.1, 120.4, 119.9, 119.7, 110.5, 99.6, 64.3, 58.6, 39.6, 36.5, 27.5, 27.2, 26.4, 25.0, 18.9, 16.3, 16.2.

HR-MS (ESI): Calculated for $C_{23}H_{31}NO$ $[M+H]^+$ 338.2479, found: 338.2485.

(3S,4aR,6aR,12aR,12bR)-4,4,6a,12b-tetramethyl-11-tosyl-1,2,3,4,4a,5,6,6a,11,12,12a,12b-dodecahydrobenzo[4,5]indeno[2,1-b]indol-3-ol (5)

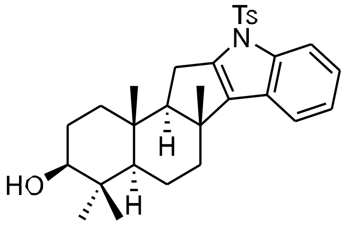

In a round-bottom flask was charged epoxide **6** (450 mg, 0.92 mmol, 1.0 eq.) and dissolved in 4.5 mL HFIP and 5 mL of CH_2Cl_2 at -18 °C. The mixture was stirred at that temperature and methanesulfonic acid (60 µL, 0.92 mmol, 1.0 eq.) were introduced dropwise. After complete addition the reaction mixture turned light brown and was complete on TLC after 15 minutes. The reaction was quenched by addition of $NaHCO_3$ solution. The reaction mixture was extracted 3x with CH_2Cl_2 and combined organic phases dried over $MgSO_4$. The crude product was purified on 25 g SiO_2 using hexanes/EA = 5/1 as eluent to give 285 mg (63% yield) of a 80:20 mixture of diastereomers (determined by ^{1}H-NMR, second diastereomer **SI-2**, depicted below). This mixture was recrystallized from heptane/toluene = 3/2 to give 169 mg (38% yield) of a single diastereomer, product **5** as a colorless solid. A single crystal for X-ray diffraction experiments was obtained by dissolving (10 mg) in $CHCl_3$, adding *n*-hexane and slowly evaporating.

m.p.: 242 °C

^{1}H NMR (600 MHz, $CDCl_3$) δ = 8.04 – 7.97 (m, 1H), 7.68 (d, *J* = 8.4, 2H), 7.32 (dt, *J* = 7.3, 0.9, 1H), 7.23 – 7.19 (m, 2H), 7.22 – 7.18 (m, 1H), 7.16 (td, *J* = 7.4, 1.2, 1H), 3.27 (dd, *J* = 11.2, 5.0, 1H), 2.98 (dd, *J* = 15.4, 6.2, 1H), 2.83 (dd, *J* = 15.4, 11.8, 1H), 2.34 (s, 3H), 2.27 (dd, *J* = 8.9, 2.9, 1H), 2.10 (dd, *J* = 11.8, 6.2, 1H), 1.86 – 1.61 (m, 6H), 1.28 – 1.17 (m, 2H), 1.10 (s, 3H), 1.06 (s, 3H), 1.01 (s, 3H), 0.99 (d, *J* = 11.8, 1H), 0.85 (s, 3H).

^{13}C NMR (151 MHz, $CDCl_3$) δ = 144.7, 141.9, 139.2, 138.0, 135.9, 130.0, 129.2, 128.4, 126.6, 126.0, 125.4, 123.3, 118.8, 114.7, 79.2, 67.1, 56.3, 43.4, 39.1, 38.6, 37.2, 37.0, 28.1, 27.2, 26.5, 21.7, 21.4, 19.2, 16.7, 15.2.

HR-MS (ESI): Calculated for $C_{30}H_{37}NO_3S$ $[M+H]^+$ 492.2567, found: 492.2568.

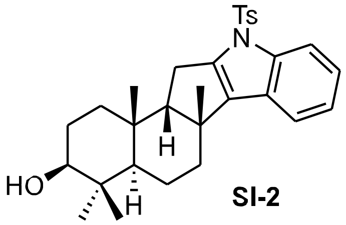

SI-2

Separation of **5** and **SI-2** was not feasible by standard column chromatography. Upon recrystallization a ca. 45:55 mixture of diastereomers (favoring **SI-2**) was obtained in the mother liquor.

Selected NMR resonances of **SI-2** from that mixture:

^{1}H NMR (600 MHz, $CDCl_3$) δ = 7.98 (d, *J* = 8.0, 1H), 7.69 (d, *J* = 8.4, 2H), 7.44 – 7.42 (m, 1H), 7.24 (d, *J* = 9.5, 2H), 3.30 (dd, *J* = 9.0, 7.0, 1H), 3.10 (dd, *J* = 16.2, 7.9, 1H), 2.98 (dd, *J* = 16.4, 10.8, 1H), 2.25 – 2.21 (m, 1H), 2.06 – 1.99 (m, 1H), 1.62 (s, 3H), 1.21 (s, 2H), 1.03 (s, 3H), 0.85 (s, 3H).

(3S,4aR,6aR,12aR,12bR)-4,4,6a,12b-tetramethyl-1,2,3,4,4a,5,6,6a,11,12,12a,12b-dodecahydrobenzo[4,5]indeno[2,1-b]indol-3-ol (12)

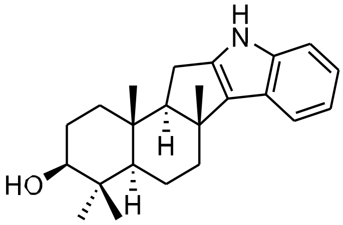

In a 25-mL round-bottom flask was charged pentacycle **5** (100 mg, 0.20 mmol, 1.0 eq.) and dissolved in methanol (10 mL). Magnesium turnings (247 mg, 10.2 mmol, 50 eq.) were added followed by solid NH_4Cl (271 mg, 5.08 mmol, 25 eq.) and the suspension was stirred before being placed in the ultrasonicator for 30 minutes. Further stirring at room temperature for 1 hour resulted in complete conversion of the starting material on TLC. The reaction was quenched by the addition of aqueous NH_4Cl solution and extracted with CH_2Cl_2 (3x 20 mL). The combined extracts were dried over $MgSO_4$ and evaporated to dryness. The crude product was purified by column chromatography using 8 g SiO_2 and hexanes/EA = 4/1 as eluent to give 65 mg (95% yield) of indole **12** as a colorless solid.

m.p.: 215 – 216 °C.

^{1}H NMR (600 MHz, $CDCl_3$) δ = 7.86 (s, 1H), 7.47 – 7.43 (m, 1H), 7.33 – 7.28 (m, 1H), 7.11 – 7.02 (m, 2H), 3.31 – 3.18 (m, 1H), 2.65 (dd, *J* = 14.2, 11.7, 1H), 2.55 (dd, *J* = 14.1, 6.1, 1H), 2.42 – 2.34 (m, 1H), 2.21 (dd, *J* = 11.7, 6.1, 1H), 1.78 (td, *J* = 12.9, 11.9, 3.2, 2H), 1.74 – 1.66 (m, 3H), 1.65 – 1.60 (m, 1H), 1.58 (dt, *J* = 13.0, 3.5, 1H), 1.24 – 1.18 (m, 1H), 1.13 (s, 3H), 1.10 (s, 3H), 1.02 (s, 3H), 1.00 (d, *J* = 2.7, 1H), 0.86 (s, 3H).

^{13}C NMR (151 MHz, $CDCl_3$) δ = 141.4, 139.3, 129.6, 123.2, 120.4, 119.5, 118.2, 111.6, 79.3, 68.3, 56.5, 43.4, 39.1, 38.7, 38.1, 37.0, 28.2, 27.2, 24.2, 22.1, 19.3, 16.7, 15.3.

HR-MS (ESI): Calculated for $C_{23}H_{31}NO$ $[M+H]^+$ 338.2479, found: 338.2484.

Greenwaylactam A, (2aR,4S,6aS,6bR,14aR)-4-hydroxy-3,3,6a,14a-tetramethyl-1,2a,3,4,5,6,6a,6b,7,14a-decahydrobenzo[b]naphtho[2,1-e]azocine-8,14(2H,9H)-dione (3)

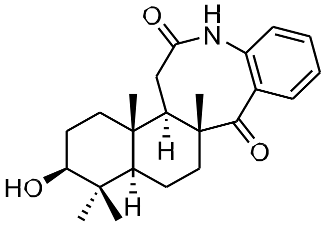

In a 25-mL round-bottom flask was charged indole **12** (30 mg, 89 μmol, 1.0 eq.) and dissolved in 3mL CH_2Cl_2. To the solution was added 1.5 mL of a saturated $NaHCO_3$ solution, followed by *m*-CPBA (77%, 50 mg, 0.22 mmol, 2.5 eq.) at 0°C. After 30 minutes at 0 °C the reaction was allowed to warm to room temperature and was stirred for 1 hour. The mixture was quenched by addition of thiosulfate solution and extracted with CH_2Cl_2 (3x 10 mL) to give crude product. Purification via flash chromatography using 3 g SiO_2 and 1 to 3% of methanol in CH_2Cl_2 to give 28 mg of greenwaylactam A (**3**, 85% yield, m.p.: 261 – 262 °C) as a white-pinkish solid. Recrystallization from MTBE afforded colorless material with an enhanced melting point of 274 °C.

m.p.: 274 °C (Lit [9]: 277 °C, Lit [8]: 287 °C).

^{1}H NMR (600 MHz, DMSO-*d*$_6$) δ = 9.68 (s, 1H), 7.38 (t, *J* = 7.8, 1H), 7.29 (app d, *J* = 7.5, 1H), 7.24 (app t, *J* = 7.6, 1H), 7.07 (d, *J* = 8.0, 1H), 4.40 (d, *J* = 5.1, 1H), 3.12 – 2.96 (m, 1H), 2.24 (dd, *J* = 13.4, 7.8, 1H), 2.13 – 2.07 (m, 1H), 2.03 (d, *J* = 13.1, 1H), 1.72 (app d, *J* = 13.5, 1H), 1.65 (app d, *J* = 13.4, 1H), 1.61 – 1.31 (m, 4H), 1.22 (s, 3H), 1.07 (m, 1H), 1.05 – 0.95 (m, 1H), 0.93 (s, 3H), 0.87 – 0.81 (m, 1H), 0.72 (s, 3H), 0.68 (s, 3H).

^{13}C NMR (151 MHz, DMSO-*d*$_6$) δ = 211.7, 173.7, 137.9, 133.0, 129.1, 126.8, 124.6 (2C), 76.5, 54.6, 54.5, 50.5, 39.1, 38.6, 37.4, 34.0, 29.2, 28.0, 26.6, 17.9, 17.5, 16.2, 15.6.

^{1}H NMR (600 MHz, CD_3OD) δ = 7.41 (t, *J* = 7.8, 1H), 7.30 (t, *J* = 7.6, 1H), 7.23 (d, *J* = 7.7, 1H), 7.16 (d, *J* = 8.0, 1H), 3.19 (t, *J* = 8.3, 1H), 2.43 (dd, *J* = 13.5, 8.0, 1H), 2.31 – 2.24 (m, 1H), 2.20 (d, *J* = 13.2, 1H), 1.90 (d, *J* = 13.2, 1H), 1.84 – 1.73 (m, 1H), 1.72 – 1.49 (m, 4H), 1.32 (s, 3H), 1.28 – 1.22 (m, 1H), 1.17 (m, 1.02 (s, 3H), 0.99 – 0.93 (m, 1H), 0.85 (s, 3H), 0.79 (s, 3H).

^{13}C NMR (151 MHz, CD_3OD) δ = 213.5, 177.5, 139.9, 133.8, 130.5, 128.0, 126.6, 126.1, 79.1, 56.6, 56.4, 52.2, 40.8, 40.0, 39.1, 35.7, 30.3, 28.5, 27.5, 19.0, 18.9, 16.9, 15.9.

HR-MS (ESI): Calculated for $C_{23}H_{31}NO_3$ $[M+H]^+$ 370.2377, found: 370.2380.

NMR comparison with original isolation report and synthesis

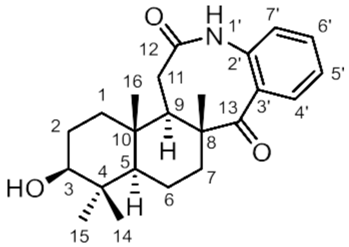

Atom	Natural **3**[9] (500 MHz, DMSO-d$_6$) δ$_H$ [ppm]	Synthetic **3**[8] (400 MHz, DMSO-d$_6$) δ$_H$ [ppm]	Synthetic **3** - this work (600 MHz, DMSO-d$_6$) δ$_H$ [ppm]	Δδ$_H$ [ppm]
**1**	1.73	1.72, 1.00	1.72, 1.01	-0.01
**2**	1.51	1.51	1.51	-
**3**	3.03, 4.38 (*J* = 5.1)	3.03, 4.36 (*J* = 5.0)	3.03, 4.40 (*J* = 5.1)	+0.02
**5**	0.84	0.85	0.84	-
**6**	1.46, 1.65	1.47, 1.66	1.47, 1.65	+0.01
**7**	1.59, 2.10	1.59, 2.11	1.59, 2.10	-
**9**	1.07 (*J* = 6.2)	1.08	1.07	-
**11**	2.02, 2.24	2.04, 2.25 (*J* = 13.3, 7.9)	2.03, 2.24 (*J* = 13.4, 7.8)	-
**14**	0.68	0.68	0.68	-
**15**	0.93	0.93	0.93	-
**16**	0.73	0.73	0.72	-0.01
**17**	1.22	1.22	1.22	-
**1’**	9.68	9.67	9.68	-
**4’**	7.29 (*J* = 7.1)	7.29	7.29 (*J* = 7.5)	-
**5’**	7.24 (t, *J* = 7.1)	7.25	7.24 (t, *J* = 7.6)	-
**6’**	7.38 (*J* = 7.1, 8.1)	7.38 (*J* = 7.3)	7.38 (*J* = 7.8)	-
**7’**	7.07 (*J* = 8.1)	7.08 (*J* = 8.0)	7.07 (*J* = 8.0)	-

Atom	Natural **3**[9] (125 MHz, DMSO-d$_6$) δ$_C$ [ppm]	Synthetic **3**[8] (101 MHz, DMSO-d$_6$) δ$_C$ [ppm]	Synthetic **3** - this work (151 MHz, DMSO-d$_6$) δ$_C$ [ppm]	Δδ$_C$ [ppm]**
**1**	37.3	37.4	37.4	+0.1
**2**	26.5	26.6	26.6	+0.1
**3**	76.3	76.4	76.5	+0.2
**4**	38.4	38.5	38.6	+0.1
**5**	54.3	54.5	54.6	+0.3
**6**	17.3	17.4	17.5	+0.2
**7**	33.9	34.0	34.0	+0.1
**8**	50.3	50.4	50.5	+0.2
**9**	54.5	54.5	54.5	-
**10**	38.9	39.0	39.1	+0.2

**11**	29.0	29.1	29.2	+0.2
**12**	173.5	173.6	173.7	+0.2
**13**	211.5	211.6	211.7	+0.2
**14**	15.5	15.6	15.6	+0.1
**15**	27.8	28.0	28.0	+0.2
**16**	16.0	16.1	16.2	+0.2
**17**	17.7	17.8	17.9	+0.2
**2’**	132.8	132.9	133.0	+0.2
**3’**	138.0	137.9	137.9	-0.1
**4’**	128.9*	126.7	126.8	+0.1
**5’**	126.6*	124.5	124.6	+0.1
**6’**	128.9*	129.0	129.1	+0.1
**7’**	126.6*	124.6	124.6	-

*Probably an error in reference [9] upon comparison with literature spectrum in the Supporting Information of [9]

**A systematic shift of +0.1ppm was already described for the ^{13}C-shifts in [8], in our case the systematic shift is between 0.1 and 0.2 ppm, biggest overall delta = 0.4 ppm (+0.3 to -0.1).

The signals of the ^{1}H and ^{13}C-NMR spectra are in good agreement except for erroneously copied shifts. All signals match with the synthesized material [8].

Greenwaylactam C, (2aR,4S,6aS,6bR,14aR)-3,3,6a,14a-tetramethyl-8,14-dioxo-1,2,2a,3,4,5,6,6a,6b,7,8,9,14,14a-tetradecahydrobenzo[b]naphtho[2,1-e]azocin-4-yl acetate (4)

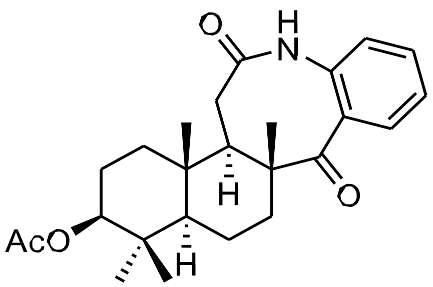

In a 10-mL round-bottom flask was charged greenwaylactam A (5.0 mg, 14 µmol, 1.0 eq.) and dissolved in dry CH_2Cl_2 (1.2 mL). At -25 °C there was added 4-DMAP (2.5 mg, 20 µmol, 1.5 eq.) followed by a stock solution of Ac_2O (4 mg, 41 µmol, 3.0 eq.) in CH_2Cl_2. The mixture was stirred at that temperature for 30 minutes and then slowly allowed to reach room temperature over 4 hours, at which point the reaction was complete by TLC analysis. The reaction mixture was directly applied onto a 0.7 g SiO_2 column and eluted with 2% methanol in CH_2Cl_2 to give 4.5 mg (81% yield) of greenwaylactam C (**4**) as a colorless solid.

m.p.: 247 – 250 °C (Lit [9]: 249 – 250 °C).

^{1}H NMR (600 MHz, $CDCl_3$) δ = 7.42 – 7.32 (m, 2H), 7.27 (m, 1H), 7.13 – 7.05 (m, 2H), 4.69 – 4.37 (m, 1H), 2.44 (m, 1H), 2.39 – 2.28 (m, 1H), 2.27 – 2.15 (m, 1H), 2.04 (s, 3H), 1.92 (m, 1H), 1.87 – 1.70 (m, 2H), 1.67 – 1.56 (m, 2H), 1.54 – 1.45 (m, 1H), 1.45 – 1.38 (m, 1H), 1.31 (s, 3H), 1.34 – 1.25 (m, 1H), 1.07 (m, 1H), 0.90 (s, 3H), 0.86 (s, 6H).

^{13}C NMR (151 MHz, $CDCl_3$) δ = 211.3, 174.7, 170.9, 138.9, 132.0, 129.7, 127.0, 125.9, 125.2, 80.2, 54.8, 38.0. 37.5, 54.3, 51.2, 23.2, 39.6, 34.6, 29.3, 28.0, 21.4, 18.9, 17.8, 16.6, 16.5.

HR-MS (ESI): Calculated for $C_{25}H_{33}NO_4$ $[M+H]^+$ 412.2483, found: 412.2489.

NMR comparison with original isolation report and synthesis

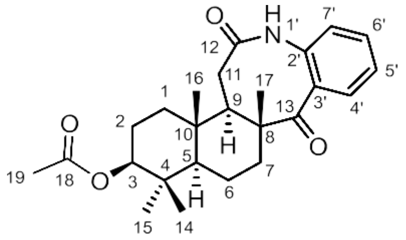

Atom	Natural **4**[9] (500 MHz, $CDCl_3$) δ_H [ppm]	Synthetic **4**[8] (600 MHz, $CDCl_3$) δ_H [ppm]	Synthetic **4** - this work (600 MHz, $CDCl_3$) δ_H [ppm]	$\Delta\delta_H$ [ppm]
**1**	1.32, 1.90	1.30, 1.91	1.29, 1.92	+0.03, +0.02
**2**	1.62, 1.77	1.60, 1.78	1.61, 1.78	+0.01, -0.01
**3**	4.49	4.50	4.51	-0.02
**5**	1.08	1.09	1.07	+0.01
**6**	1.49, 1.75	1.50, 1.77	1.50, 1.77	-0.01, -0.02
**7**	1.62, 1.65*	1.63 (J = 13.4, 2.9), 2.34,	1.63, 2.33	-0.01, -0.69
**9**	1.44	1.42	1.42	+0.02
**11**	2.44*	2.22, 2.44	2.21, 2.44	+0.22
**14**	0.87	0.86	0.86	+0.01
**15**	0.91	0.90	0.90	+0.01
**16**	0.87	0.86	0.86	+0.01
**17**	1.32	1.31	1.31	+0.01
**19**	2.04	2.04	2.04	-
**1’**	7.61	7.20	7.38	+0.23
**4’**	7.10 (*J* = 7.2)	7.11	7.11	-0.01
**5’**	7.28 (*J* = 7.2)	7.28	7.27	+0.01
**6’**	7.37(*J* = 7.2)	7.38	7.37	-
**7’**	7.11 (*J* = 7.2)	7.09	7.08	+0.03

Atom	Natural **4**[9] (125 MHz, $CDCl_3$) δ_C [ppm]	Synthetic **4**[8] (101 MHz, $CDCl_3$) δ_C [ppm]	Synthetic **4** - this work (151 MHz, $CDCl_3$) δ_C [ppm]	$\Delta\delta_C$ [ppm]
**1**	37.3	37.5	37.5	-0.2
**2**	23.1	23.2	23.2	-0.1
**3**	80.1	80.2	80.2	-0.1
**4**	37.8	38.0	38.0	-0.2
**5**	54.7	54.8	54.8	-0.1
**6**	17.7	17.9	17.8	-0.1
**7**	34.5	34.6	34.6	-0.1
**8**	51.2	51.3	51.2	-

**9**	54.2	54.4	54.3	-0.1
**10**	37.8*	39.7	39.6	-1.8
**11**	29.3	29.4	29.3	-
**12**	174.6	174.6	174.7	-0.1
**13**	211.2	211.3	211.3	-0.1
**14**	16.3	16.7	16.6	-0.3
**15**	27.8	28.0	28.0	-0.2
**16**	16.3	16.5	16.5	-0.2
**17**	18.7	18.9	18.9	-0.2
**18**	170.7	170.9	170.9	-0.2
**19**	21.2	21.4	21.4	-0.2
**2’**	132.0	132.0	132.0	-
**3’**	138.7	139.0	138.9	-0.2
**4’**	126.7	127.0	127.0	-0.3
**5’**	125.7	126.0	125.9	-0.2
**6’**	129.5	129.7	129.7	-0.2
**7’**	125.1	125.2	125.2	-0.1

*Probably an error in reference [9] upon comparison with literature spectrum in the Supporting Information of [9]

The signals of the ^{1}H and ^{13}C-NMR spectra are in good agreement except for erroneously copied shifts. All signals match with the synthesized material [8].

Greenwaylactam B, (2aR,6aS,6bR,14aR)-3,3,6a,14a-tetramethyl-1,2a,5,6,6a,6b,7,14a-octahydrobenzo[b]naphtho[2,1-e]azocine-4,8,14(2H,3H,9H)-trione (2)

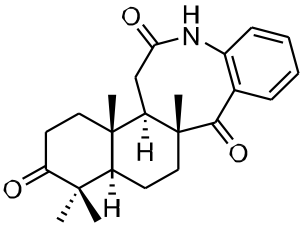

In a 10-mL round-bottom flask was charged greenwaylactam A (5.0 mg, 14 µmol, 1.0 eq.) and dissolved in dry CH_2Cl_2 (1.4 mL). At 0 °C there was added homemade and recrystallized Dess-Martin periodinane (99%, 8.6 mg, 20 µmol, 1.5 eq.) in CH_2Cl_2. The mixture was stirred at that temperature for 30 minutes and then slowly allowed to reach room temperature over 4 hours, at which point the reaction was complete by TLC analysis. The reaction mixture was directly applied onto a 0.7 g SiO_2 column and eluted with 2% methanol in CH_2Cl_2 to give 4.0 mg (80% yield) of greenwaylactam B (**2**) as a colorless solid.

m.p.: 301 °C

^{1}H NMR (600 MHz, $CDCl_3$) δ = 7.39 (t, *J* = 7.5, 1H), 7.36 – 7.32 (br s, 1H), 7.29 (t, *J* = 7.5, 1H), 7.17 – 7.06 (m, 2H), 2.56 (m, 1H), 2.53 – 2.45 (m, 2H), 2.42 – 2.32 (m, 1H), 2.23 (d, *J* = 13.3, 1H), 2.07 (m, 1H), 1.81 (m, 1H), 1.73 (d, *J* = 12.3, 1H), 1.67 (dt, *J* = 13.6, 3.0, 1H), 1.66 – 1.52 (m, 3H), 1.33 (s, 3H), 1.14 (s, 3H), 1.06 (s, 3H), 0.91 (s, 3H).

^{13}C NMR (151 MHz, $CDCl_3$) δ = 216.9, 211.1, 174.5, 138.8, 131.9, 129.8, 127.0, 126.1, 125.3, 53.8, 53.6, 51.1, 47.3, 39.2, 38.2, 34.0, 33.7, 29.7, 27.1, 20.9, 19.3, 18.3, 16.7.

HR-MS (ESI): Calculated for $C_{23}H_{29}NO_3$ $[M+H]^+$ 368.2220, found: 368.2223.

NMR comparison with original isolation report and synthesis

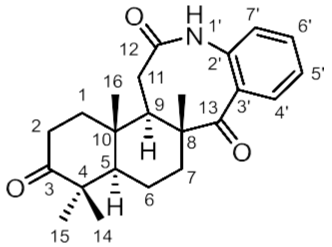

Atom	Natural **2**[9] (500 MHz, $CDCl_3$) δ_H [ppm]	Synthetic **2**[8] (700 MHz, $CDCl_3$) δ_H [ppm]	Synthetic **2** – this work (600 MHz, $CDCl_3$) δ_H [ppm]	$\Delta\delta_H$ [ppm]
**1**	1.80, 2.07	1.81, 2.07	1.81, 2.07	-0.01
**2**	2.50*	2.23, 2.49	2.23, 2.49	+0.27, 0.01
**5**	1.65	1.67	1.67 (*J* = 13.6, 3.0)	-0.02
**6**	1.61, 1.73	1.61, 1.73	1.61, 1.73 (*J* = 12.3)	-
**7**	1.67, 1.70*	1.68, 2.37	1.66, 2.37	-0.01, -0.67
**9**	1.55	1.53	1.55	-
**11**	2.52*	2.50, 2.57	2.56, 2.51	+0.02, -0.05
**14**	1.07	1.06	1.06	+0.01
**15**	1.15	1.14	1.14	+0.01
**16**	0.93	0.91	0.91	+0.02
**17**	1.35	1.33	1.33	+0.02
**1’**	7.67	7.21	7.34	+0.33
**4’**	7.12 (*J* = 7.5)	7.12 (*J* = 8.0)	7.12	-
**5’**	7.30 (*J* = 7.5)	7.30	7.29 (*J* = 7.5)	+0.01
**6’**	7.40 (*J* = 7.5)	7.39	7.39 (*J* = 7.5)	+0.01
**7’**	7.14 (*J* = 7.5)	7.11	7.14	-

Atom	Natural **2**[9] (125 MHz, $CDCl_3$) δ_C [ppm]	Synthetic **2**[8] (176 MHz, $CDCl_3$) δ_C [ppm]	Synthetic **2** - this work (151 MHz, $CDCl_3$) δ_C [ppm]	$\Delta\delta_C$ [ppm]**
**1**	38.0	38.2	38.2	-0.2
**2**	33.5*	29.7	29.7	+3.8
**3**	216.7	216.9	216.9	-0.2
**4**	47.1	47.3	47.3	-0.2
**5**	53.7	53.8	53.8	-0.1
**6**	19.2	19.3	19.3	-0.1
**7**	33.9	34.0	34.0	-0.1
**8**	51.0	51.1	51.1	-0.1
**9**	53.5	53.7	53.6	-0.1
**10**	39.2	39.3	39.2	-
**11**	33.5	33.7	33.7	-0.2

**12**	174.4	174.4	174.5	-0.1
**13**	211.0	211.1	211.1	-0.1
**14**	20.8	20.9	20.9	-0.1
**15**	26.9	27.2	27.1	-0.2
**16**	16.5	16.7	16.7	-0.2
**17**	18.1	18.3	18.3	-0.2
**2’**	131.8	131.9	131.9	-0.1
**3’**	138.5	138.8	138.8	-0.3
**4’**	126.8	127.0	127.0	-0.2
**5’**	125.9	126.1	126.1	-0.2
**6’**	129.6	129.8	129.8	-0.2
**7’**	125.1	125.3	125.3	-0.2

*Probably an error in reference [9] upon comparison with literature spectrum in the Supporting Information of [9]

**A systematic shift of -0.2 ppm was already described in [8], in our case the systematic shift is between -0.1 and -0.2 ppm, biggest overall delta = 0.3 ppm (-0.3 to 0).

The signals of the ^{1}H and ^{13}C-NMR spectra are in good agreement except for erroneously copied shifts. All signals match with the synthesized material [8].

References:

[1] J. Koziskova, F. Hahn, J. Richter, J. Kozisek, *Acta Chim. Slov*. **2016**, 9, 136.

[2] G. M. Sheldrick, *Acta Crystallogr*. **2015**, A71, 3.

[3] G. M. Sheldrick, *Acta Crystallogr*. **2015**, C71, 3.

[4] K. D. Reichl, N. L. Dunn, N. J. Fastuca, A. T. Radosevich, *J. Am. Chem. Soc*., **2015**, 16, 5292.

[5] X. Chen, C. Pei, B. Liu, J. Li, D. Zou, Y. Wu, Y. Wu, *Chem. Commun.* **2022**, 58, 62, 8674.

[6] J. Bock, C. G. Daniliuc, U. Hennecke, *Org. Lett.,* **2019**, 21, 1704.

[7] C. Mirand, M. Döé de Maindreville, D. Cartier, J. Lévy, *Tetrahedron Lett.* **1987**, 28, 3565.

[8] I. Plangger, T. Pinkert, K. Wurst, T. Magauer, *Angew. Chem. Int. Ed.*, **2023**, e202307719.

[9] M. F. Kemgni, L. B. N. Chenda, J. Tchamgoue, P. T. Kenfack, Y. A. T. Ngandjui, S. C. N. Wouamba, G. L. M. Tiani, I. R. Green and S. F. Kouam, *ChemistrySelect,* **2021**, 6, 1705.

Spectra

(2*E*,6*E*)-1-Bromo-3,7,11-trimethyl-2,6,10-dodecatriene (8)

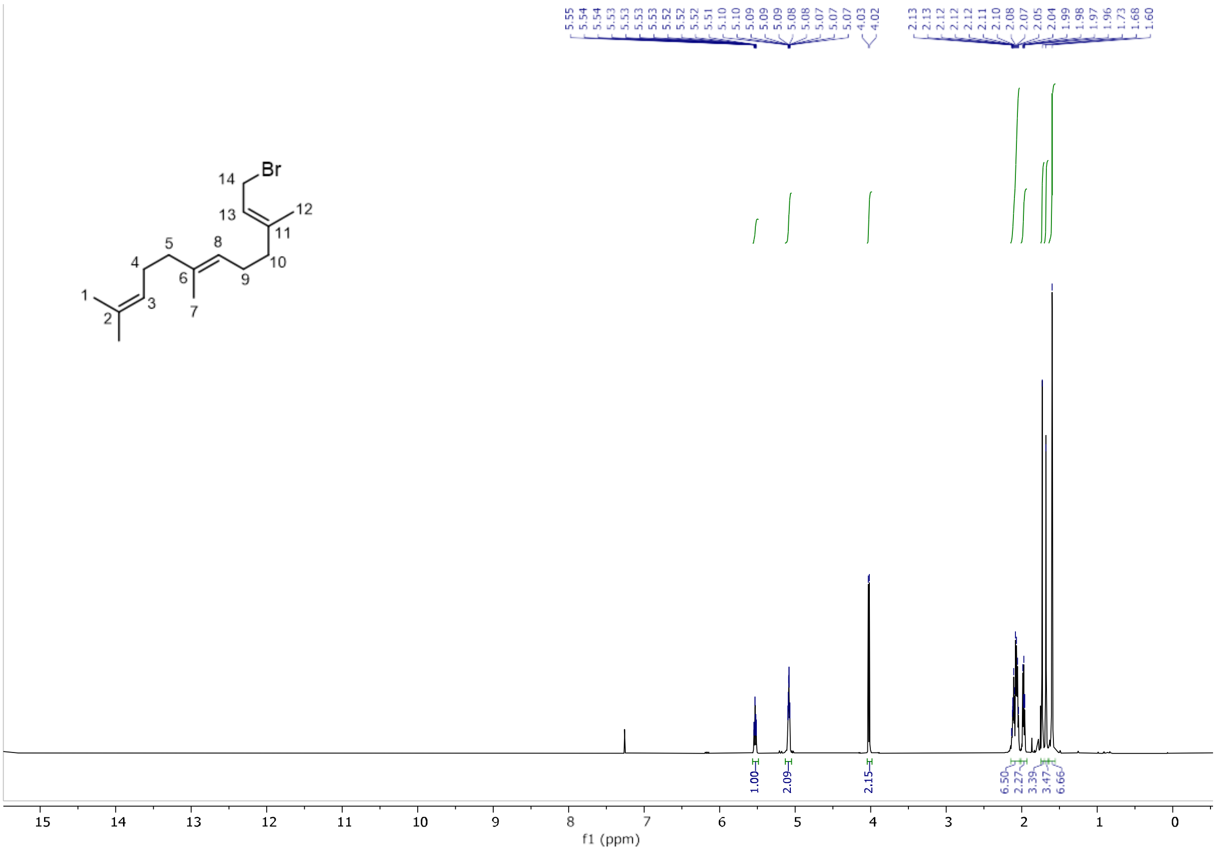

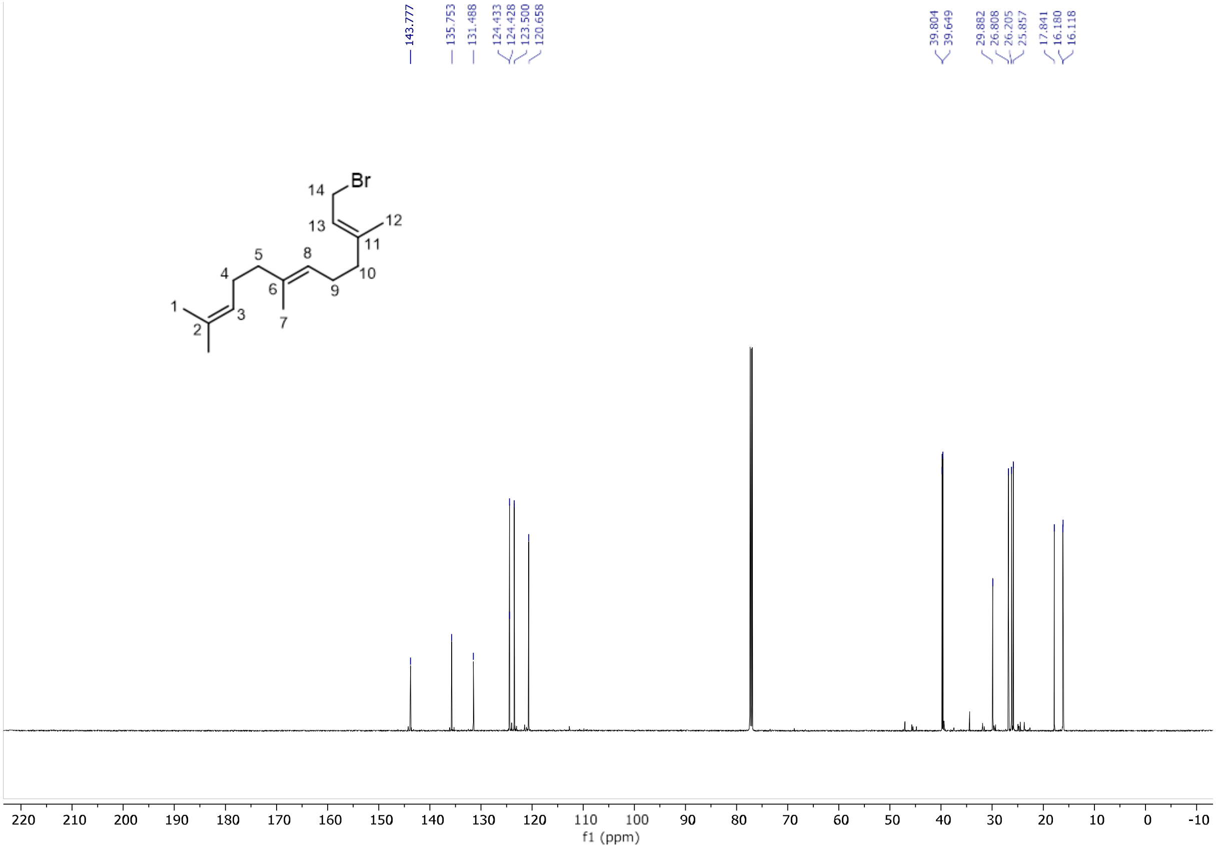

1-(*p*-Toluenesulfonyl)indole (10)

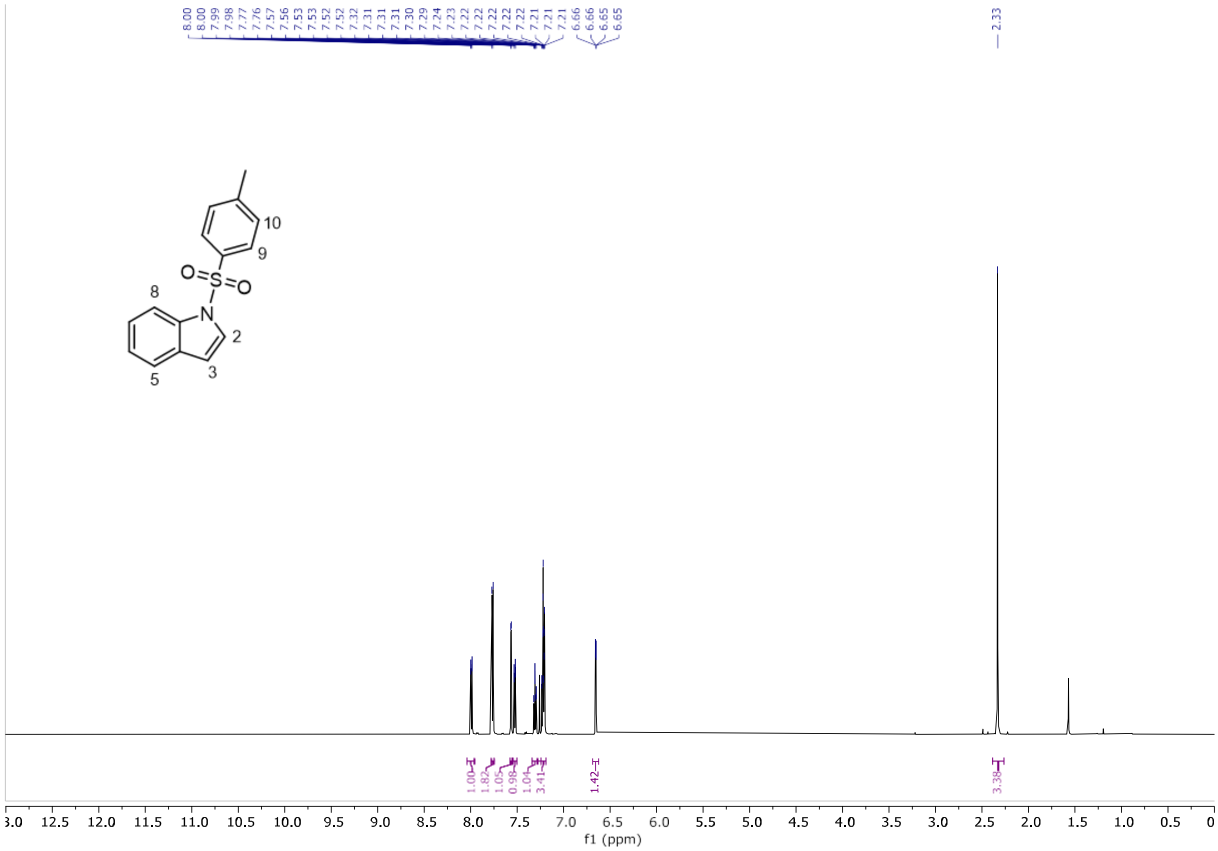

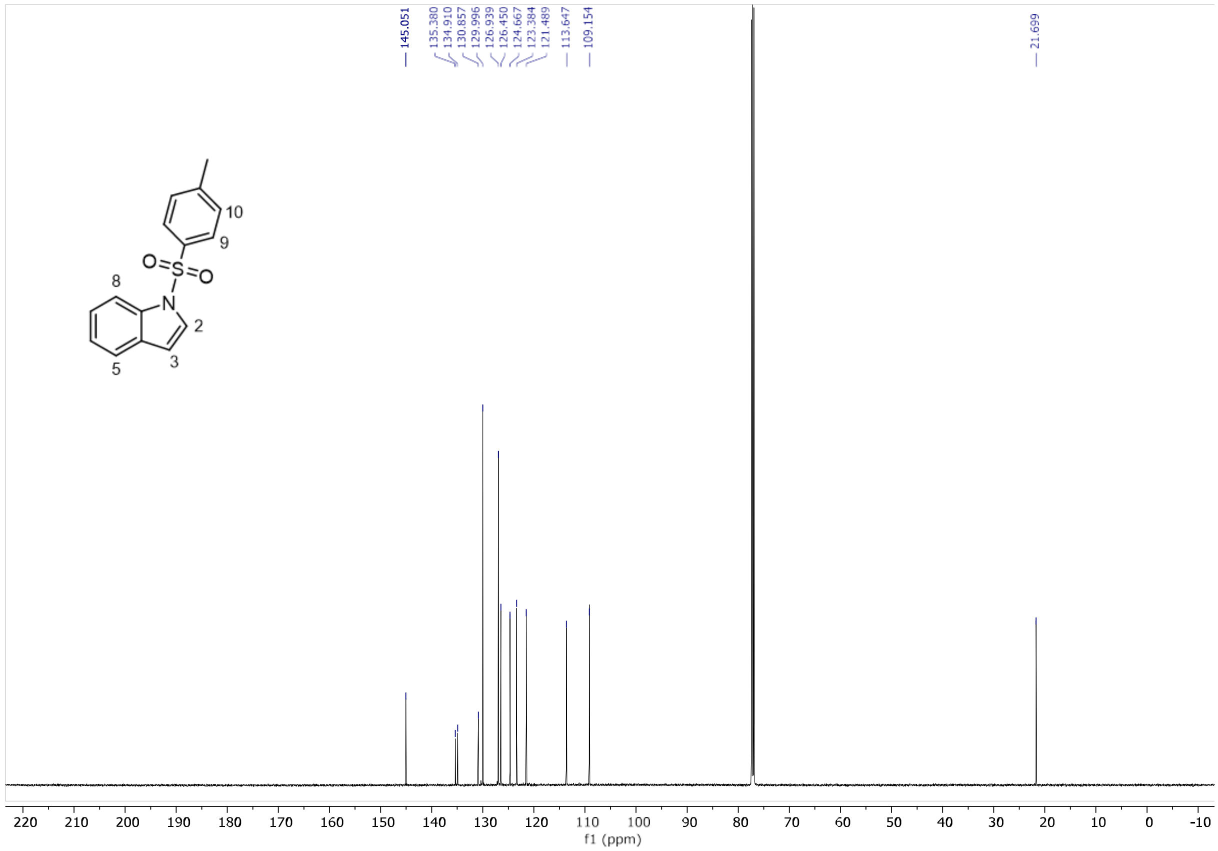

1-Tosyl-2-((2*E*,6*E*)-3,7,11-trimethyldodeca-2,6,10-trien-1-yl)-1H-indole (11)

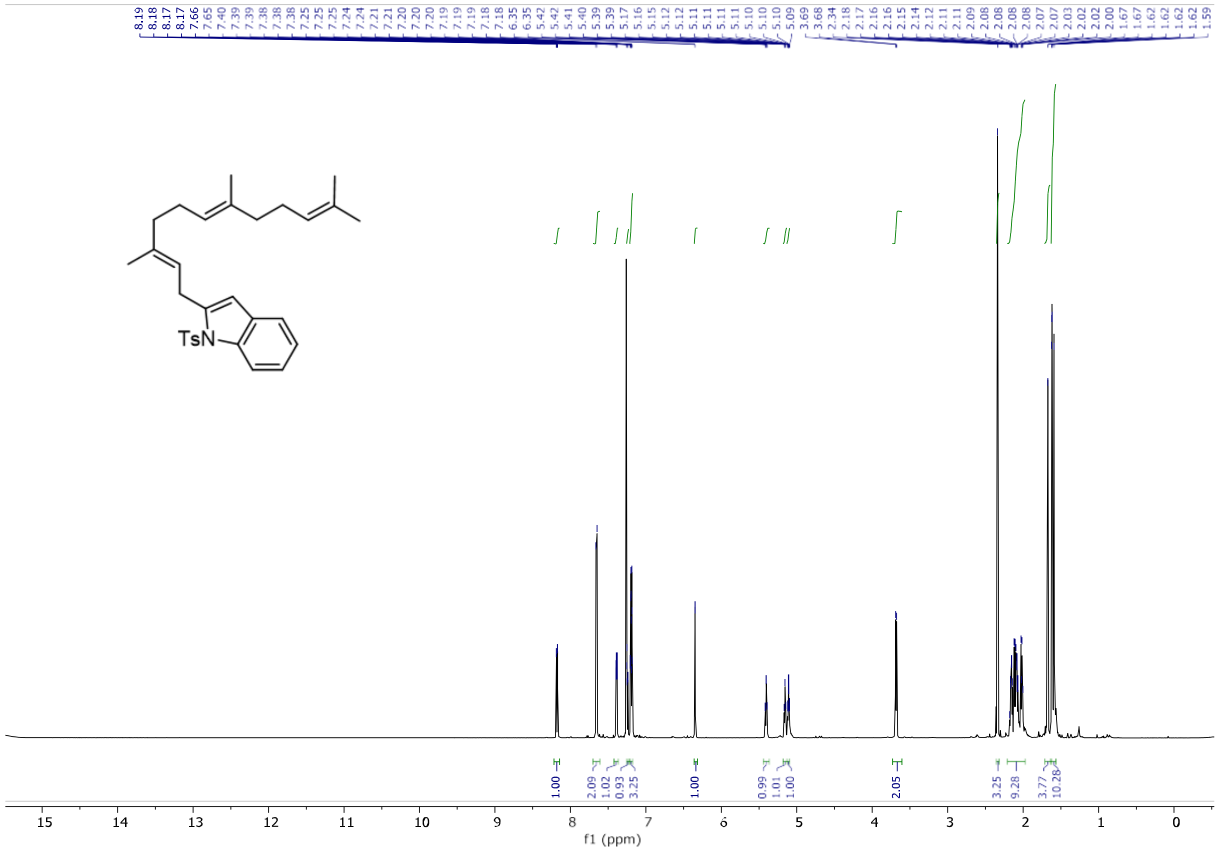

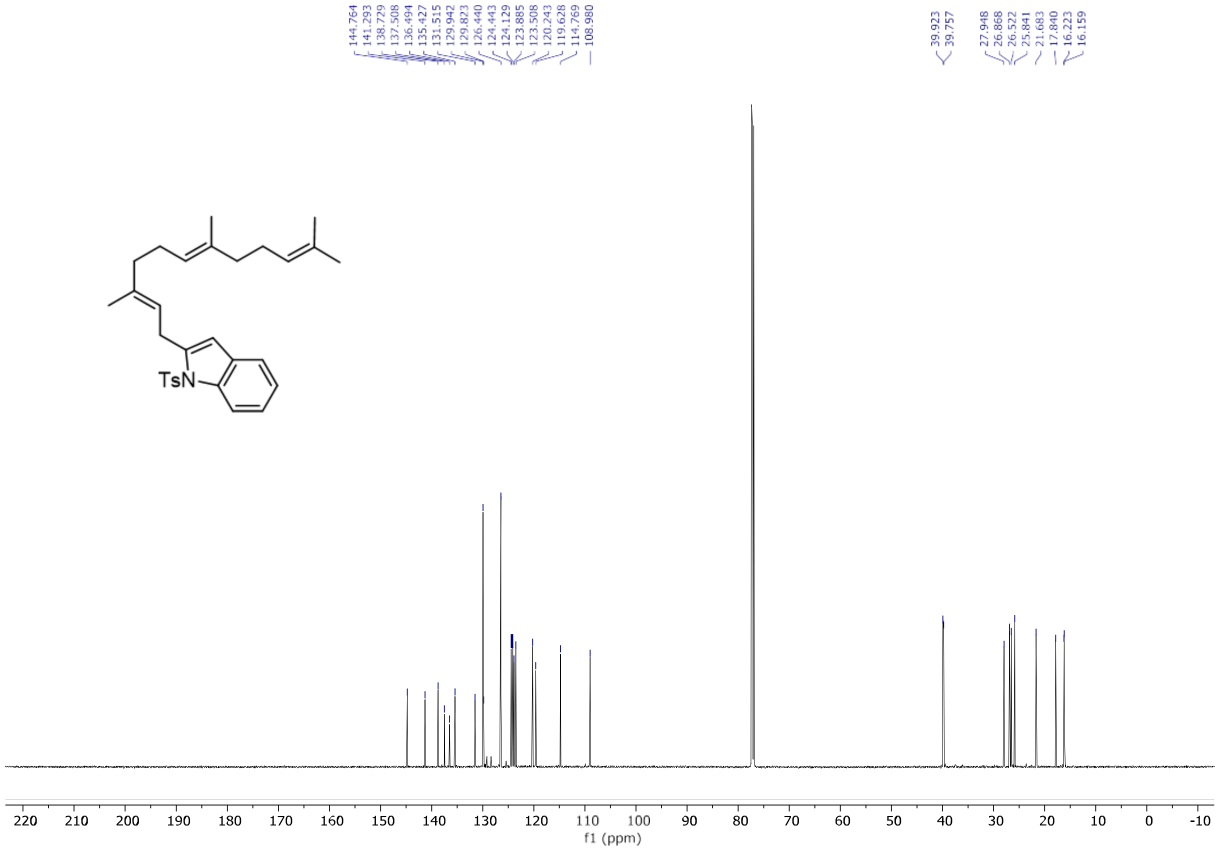

2-((2*E*,6*E*)-9-(3,3-dimethyloxiran-2-yl)-3,7-dimethylnona-2,6-dien-1-yl)-1-tosyl-1H-indole (6)

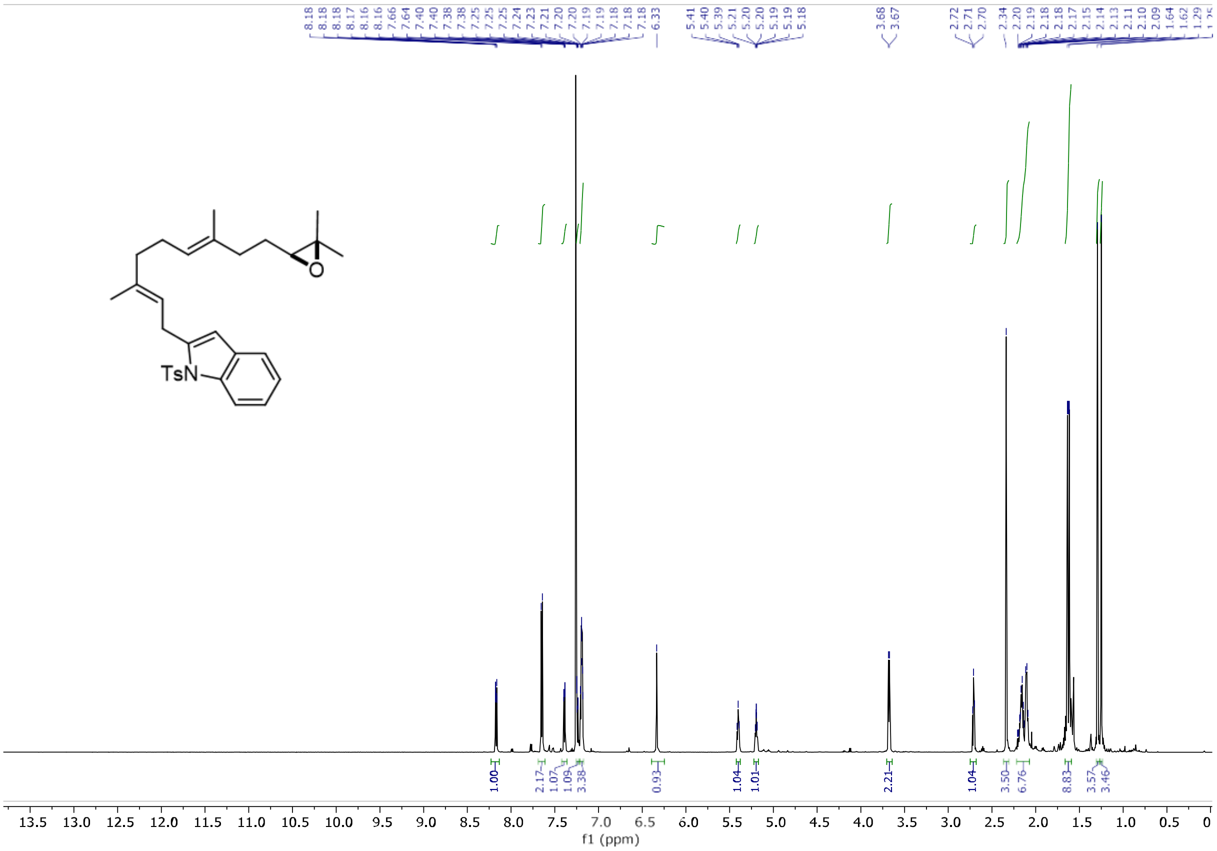

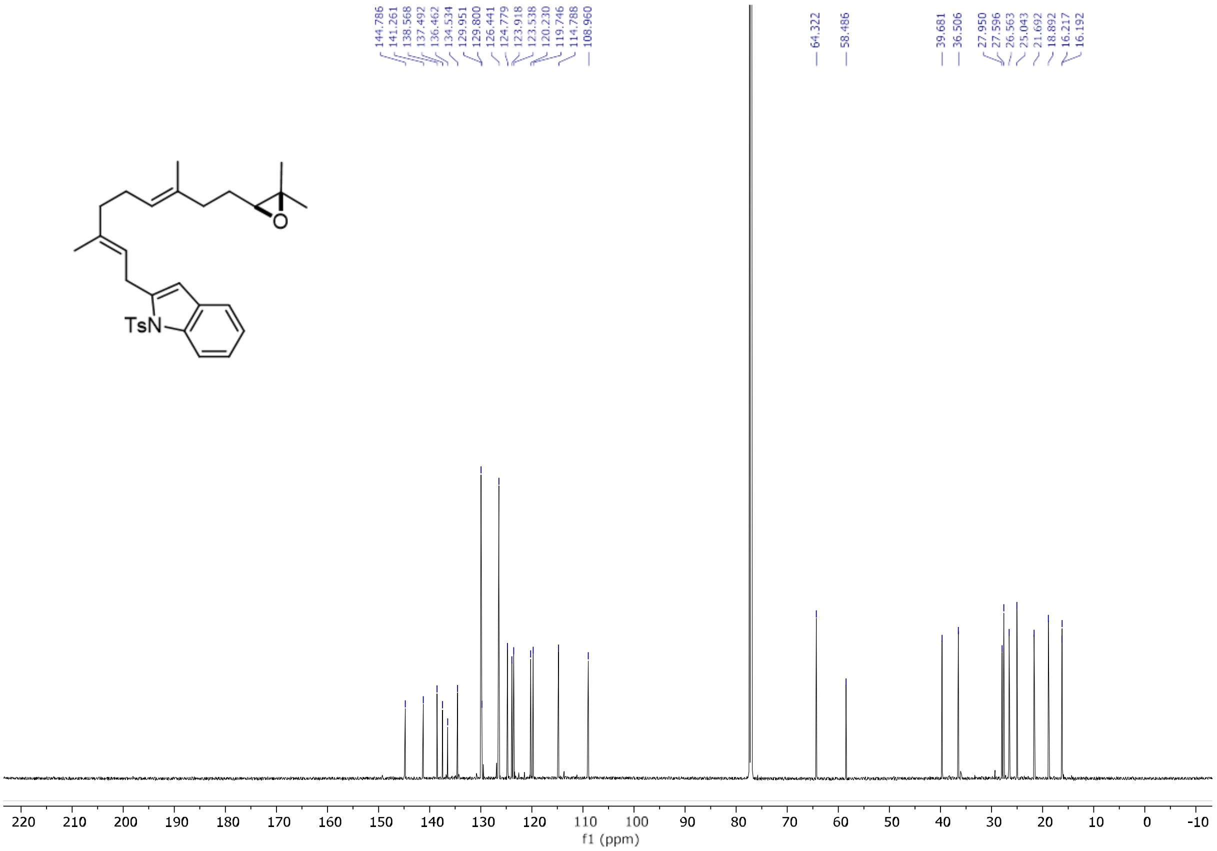

(3S,4aR,6aR,12aR,12bR)-4,4,6a,12b-tetramethyl-11-tosyl-1,2,3,4,4a,5,6,6a,11,12,12a,12b-dodecahydrobenzo[4,5]indeno[2,1-b]indol-3-ol (5)

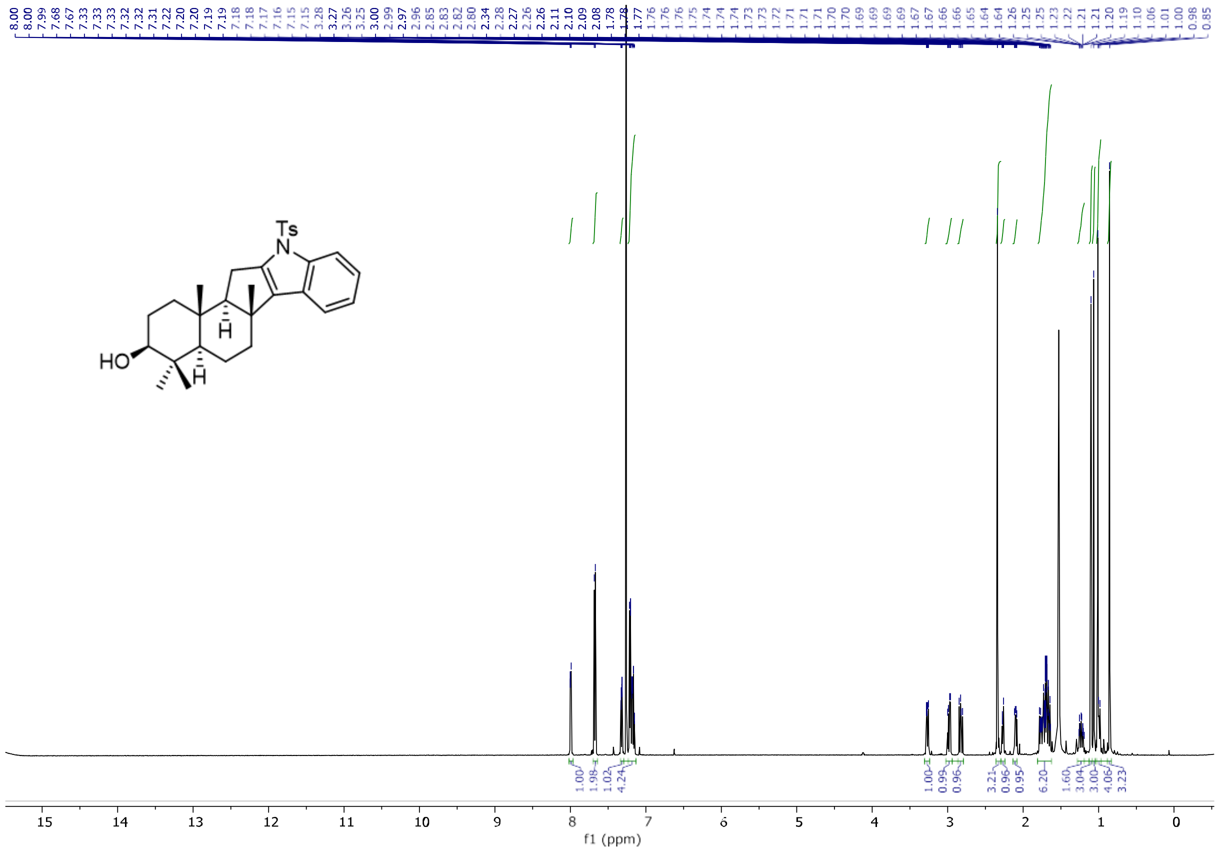

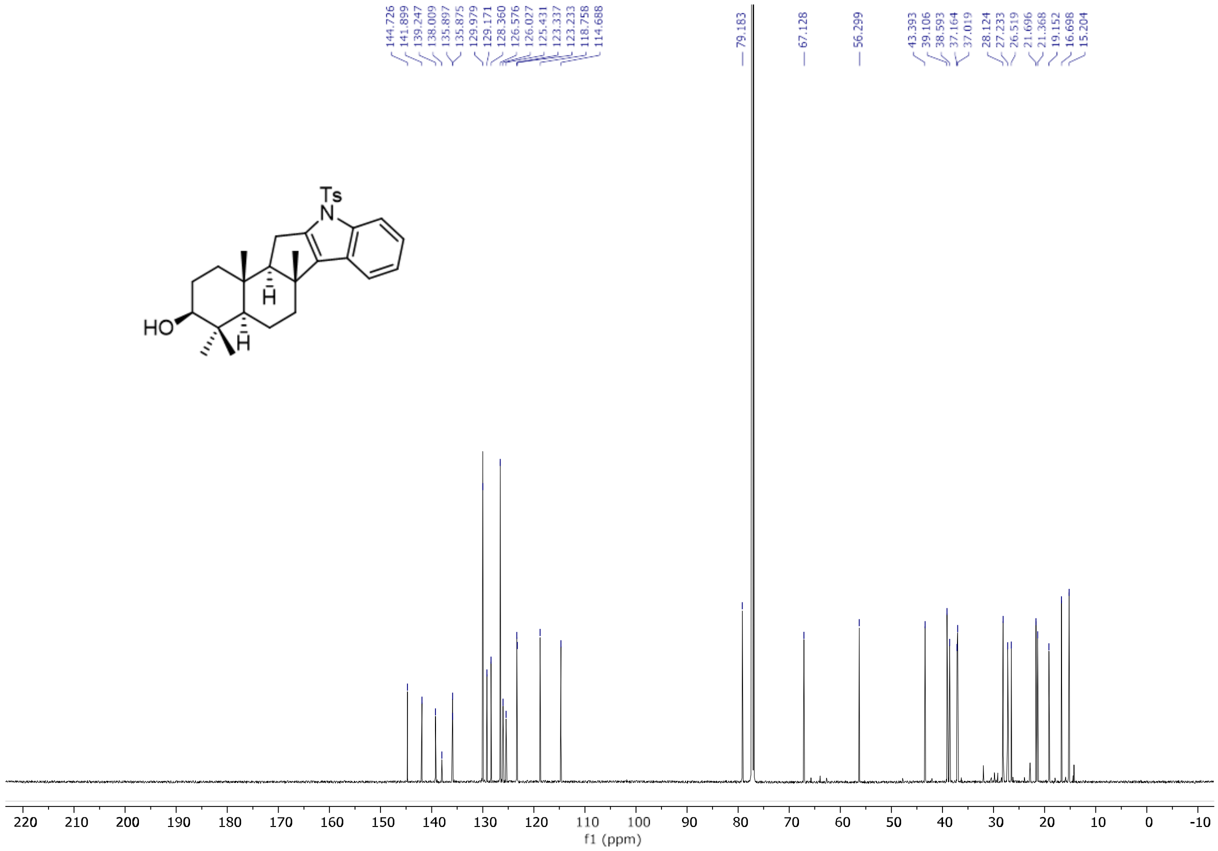

2-((2*E*,6*E*)-9-(3,3-dimethyloxiran-2-yl)-3,7-dimethylnona-2,6-dien-1-yl)-1-tosyl-1H-indole (13)

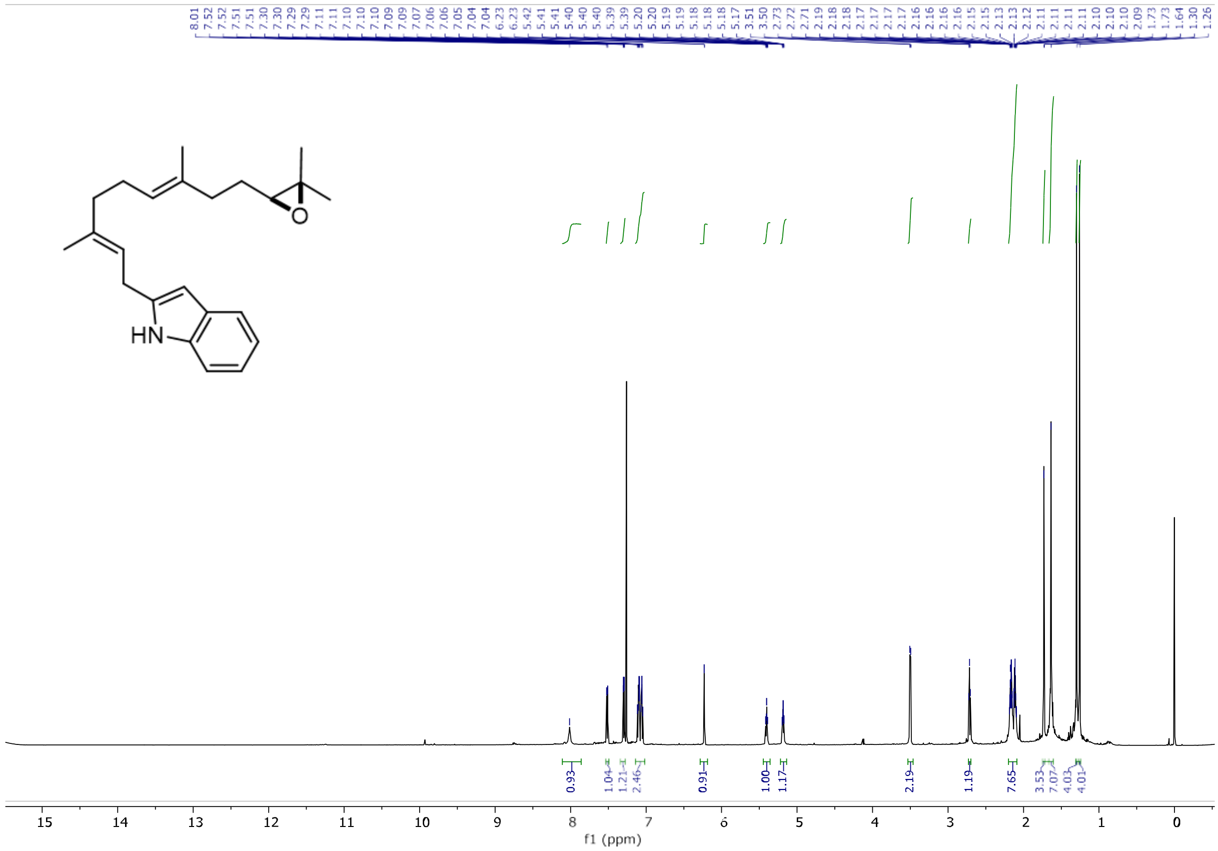

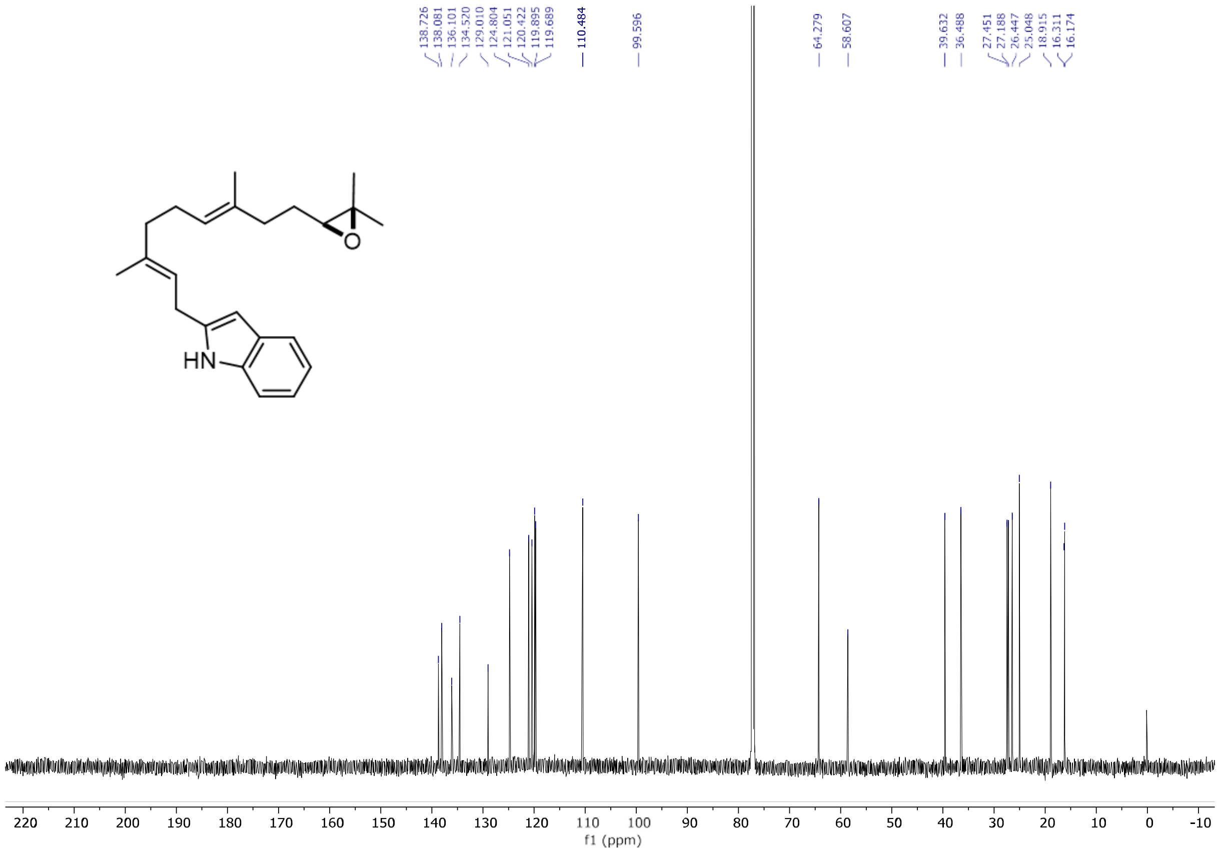

4,4,6a,12b-tetramethyl-1,2,3,4,4a,5,6,6a,11,12,12a,12b-dodecahydrobenzo[4,5]indeno[2,1-b]indol-3-ol (12)

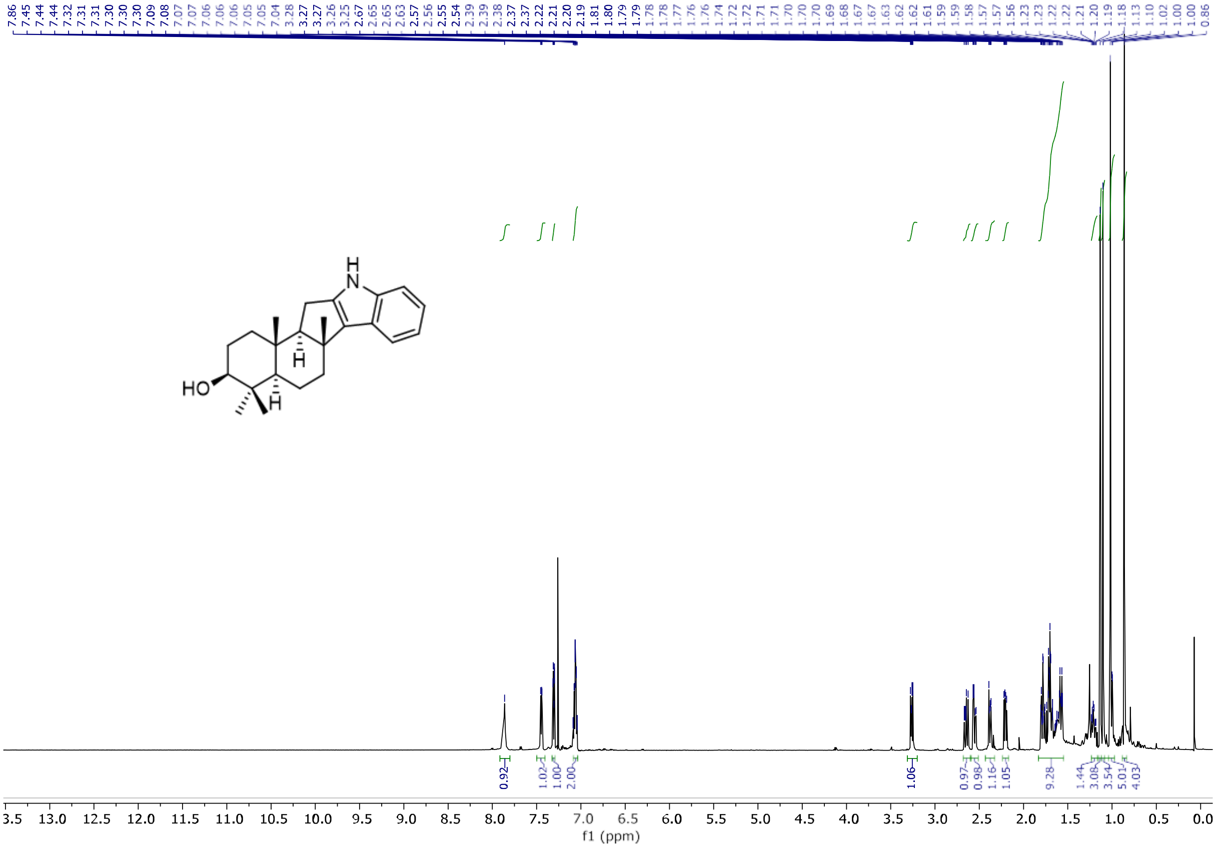

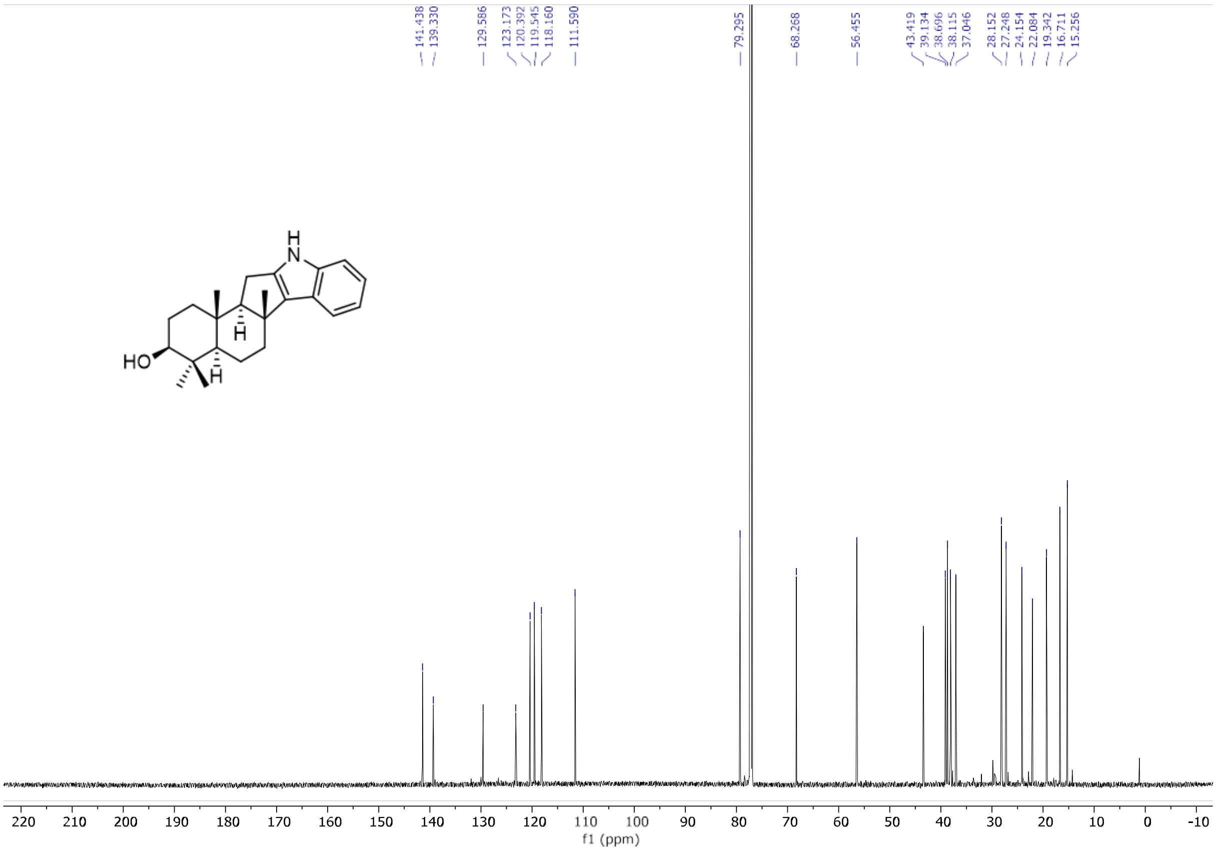

Greenwaylactam A, 4-hydroxy-3,3,6a,14a-tetramethyl-1,2a,3,4,5,6,6a,6b,7,14a-decahydrobenzo[b]naphtho[2,1-e]azocine-8,14(2H,9H)-dione (3)

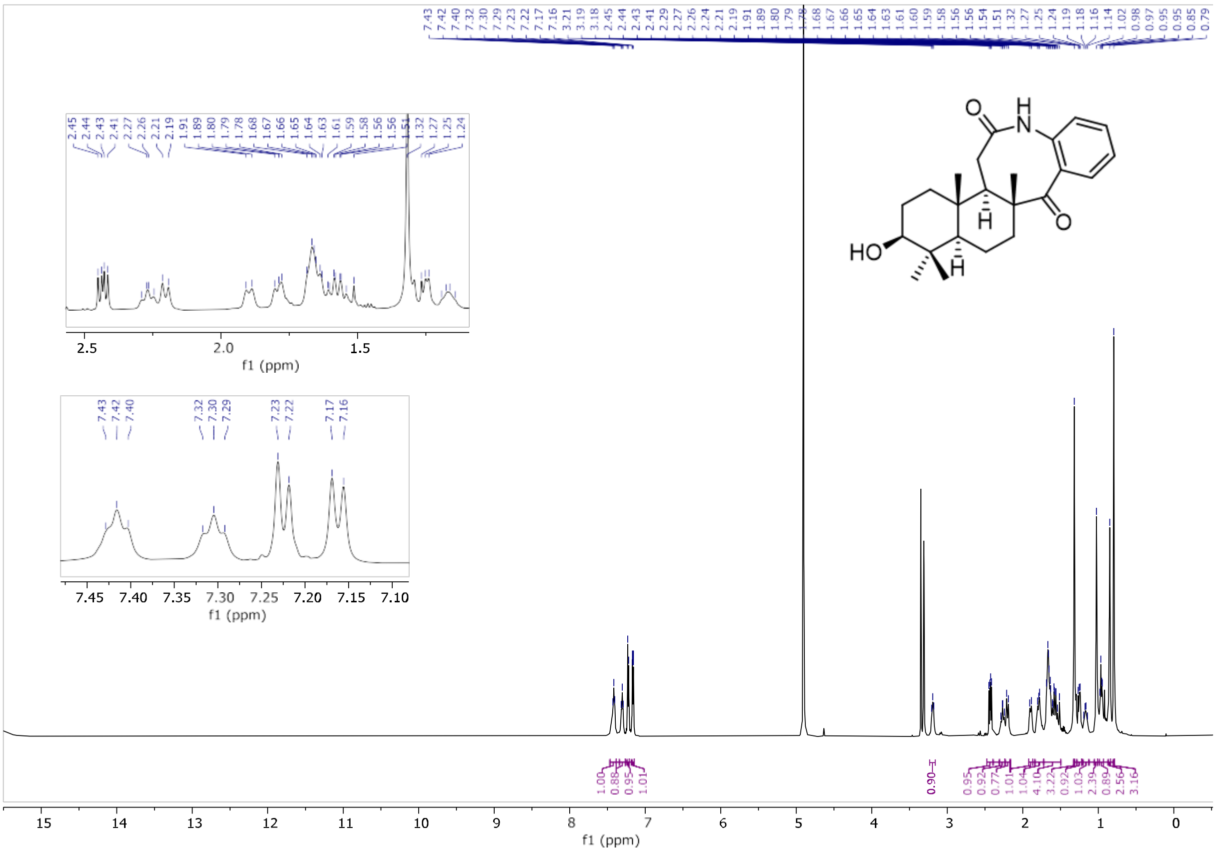

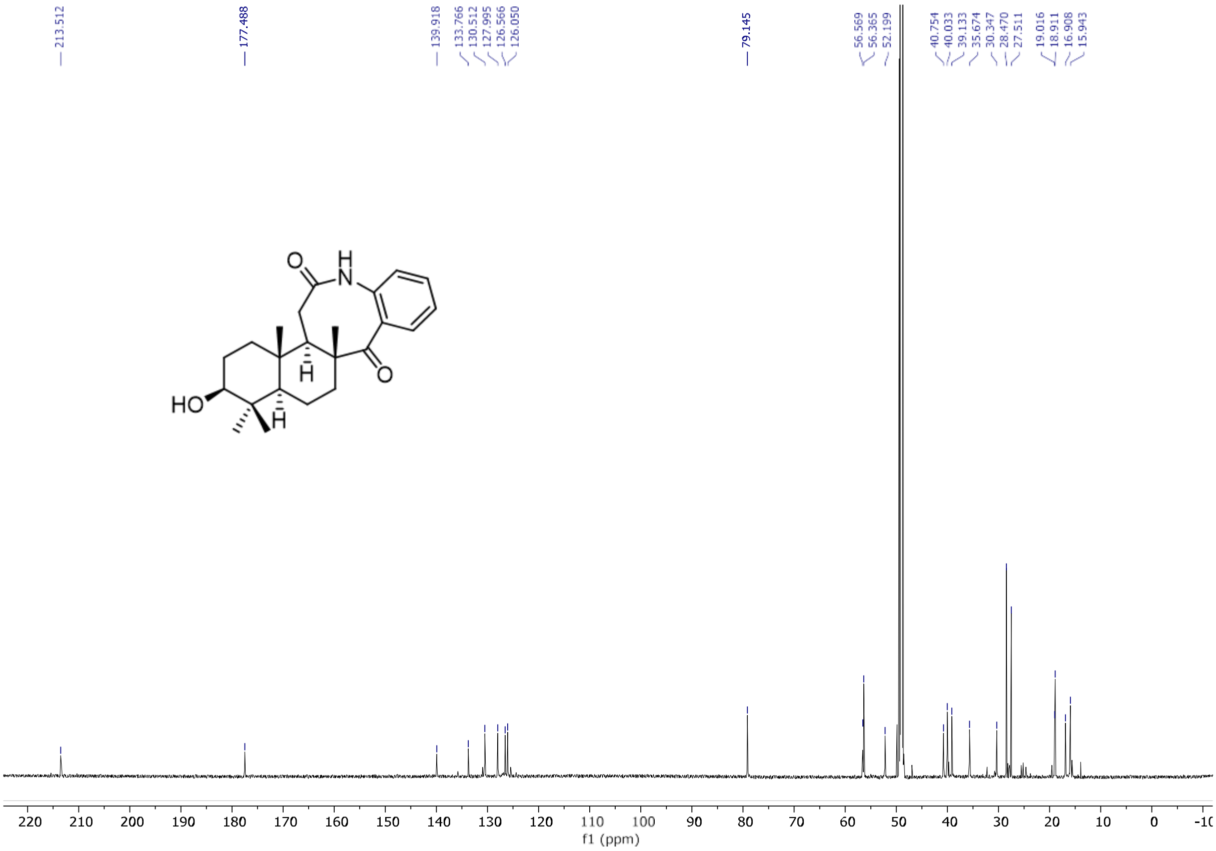

Greenwaylactam C, 3,3,6a,14a-tetramethyl-8,14-dioxo-1,2,2a,3,4,5,6,6a,6b,7,8,9,14,14a-tetradecahydrobenzo[b]naphtho[2,1-e]azocin-4-yl acetate (4)

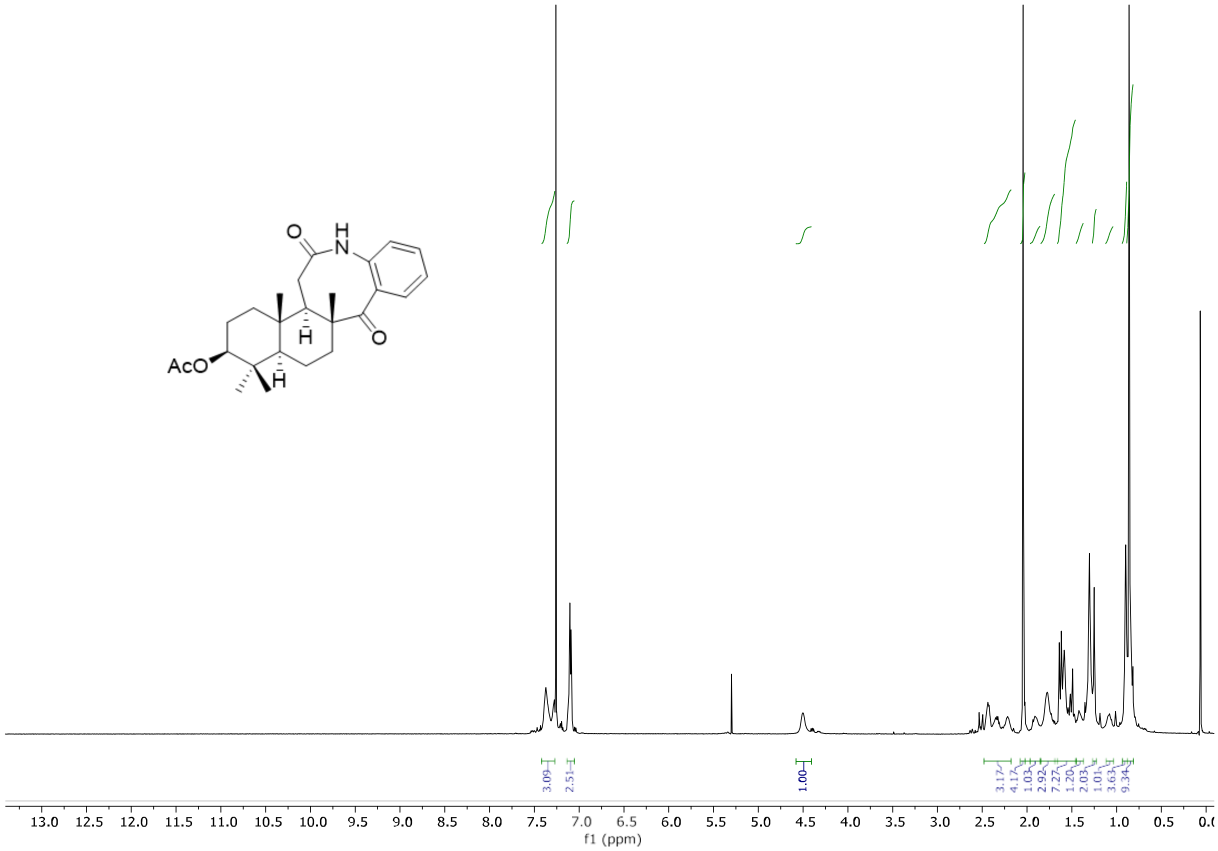

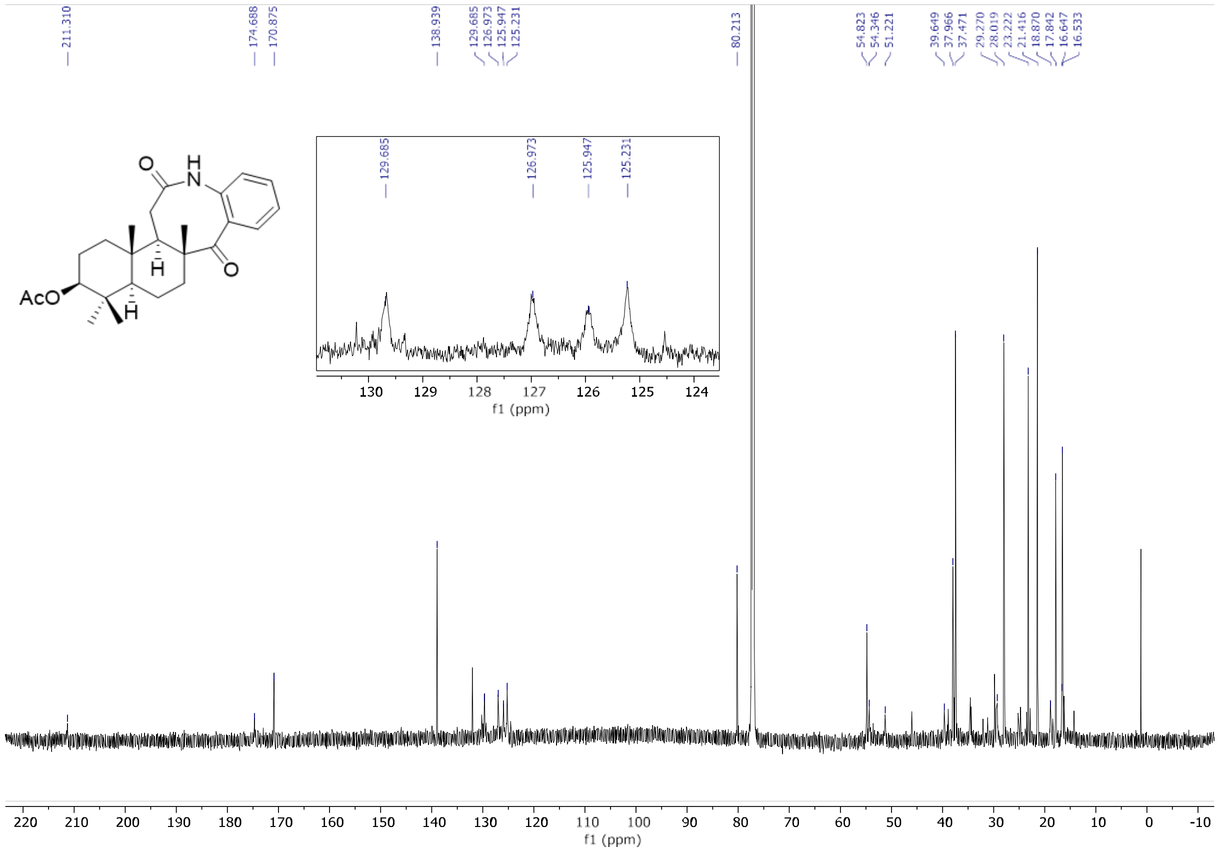

Greenwaylactam B, 3,3,6a,14a-tetramethyl-1,2a,5,6,6a,6b,7,14a-octahydrobenzo[b]naphtho[2,1-e]azocine-4,8,14(2H,3H,9H)-trione (2)

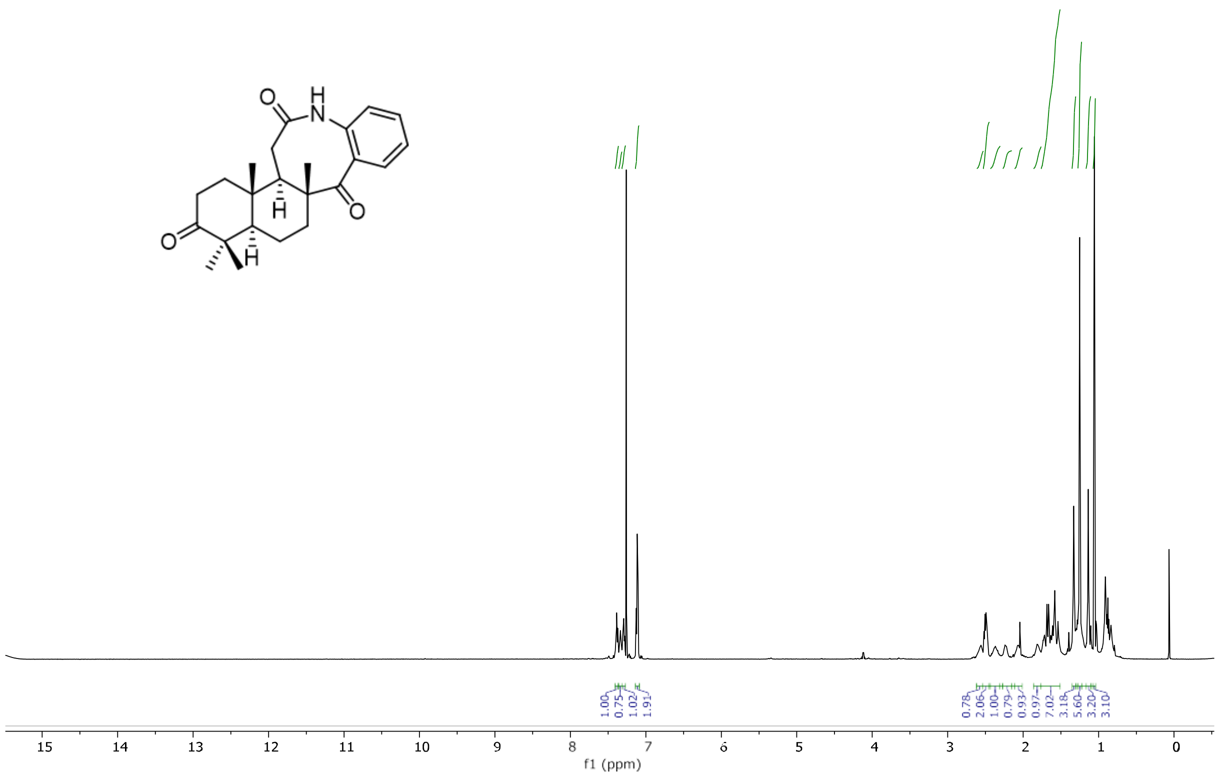

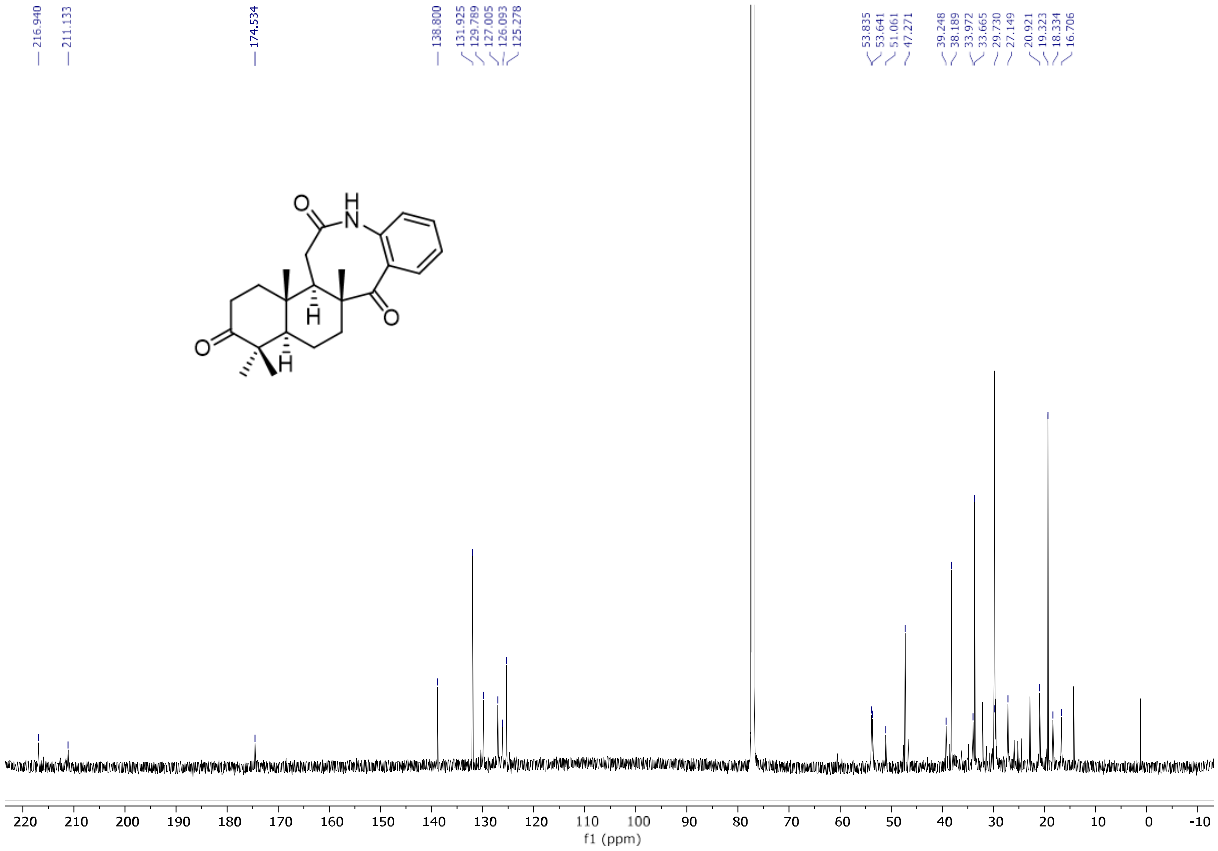

Supplement: OB-021-D3OB01001E-s001 [file OB-021-D3OB01001E-s001.pdf]
